# Proteomic and *in silico* analyses of dextran synthesis influence on *Leuconostoc lactis* AV1n adaptation to temperature change

**DOI:** 10.3389/fmicb.2022.1077375

**Published:** 2023-01-11

**Authors:** Norhane Besrour-Aouam, Vivian de Los Rios, Annel M. Hernández-Alcántara, Mᵃ Luz Mohedano, Afef Najjari, Paloma López, Hadda-Imene Ouzari

**Affiliations:** ^1^Centro de Investigaciones Biológicas Margarita Salas, CIB-CSIC, Madrid, Spain; ^2^Laboratoire Microorganismes et Biomolécules Actives (LR03ES03), Faculté des Sciences de Tunis, Université Tunis El Manar, Tunis, Tunisia

**Keywords:** *Leuconostoc*, dextran, exopolysaccharides, lactic acid bacteria, proteomic

## Abstract

*Leuconostoc lactis* is found in vegetables, fruits, and meat and is used by the food industry in the preparation of dairy products, wines, and sugars. We have previously demonstrated that the dextransucrase of *Lc. lactis* (DsrLL) AV1n produces a high-molecular-weight dextran from sucrose, indicating its potential use as a dextran-forming starter culture. We have also shown that this bacterium was able to produce 10-fold higher levels of dextran at 20°C than at 37°C, at the former temperature accompanied by an increase in *dsrLL* gene expression. However, the general physiological response of *Lc. lactis* AV1n to cold temperature in the presence of sucrose, leading to increased production of dextran, has not been yet investigated. Therefore, we have used a quantitative proteomics approach to investigate the cold temperature-induced changes in the proteomic profile of this strain in comparison to its proteomic response at 37°C. In total, 337 proteins were found to be differentially expressed at the applied significance criteria (adjusted *p*-value ≤ 0.05, FDR 5%, and with a fold-change ≥ 1.5 or ≤ 0.67) with 204 proteins overexpressed, among which 13% were involved in protein as well as cell wall, and envelope component biosynthesis including DsrLL. Proteins implicated in cold stress were expressed at a high level at 20°C and possibly play a role in the upregulation of DsrLL, allowing the efficient synthesis of the protein essential for its adaptation to cold. Post-transcriptional regulation of DsrLL expression also seems to take place through the interplay of exonucleases and endonucleases overexpressed at 20°C, which would influence the half-life of the *dsrLL* transcript. Furthermore, the mechanism of cold resistance of *Lc. lactis* AV1n seems to be also based on energy saving through a decrease in growth rate mediated by a decrease in carbohydrate metabolism and its orientation toward the production pathways for storage molecules. Thus, this better understanding of the responses to low temperature and mechanisms for environmental adaptation of *Lc. lactis* could be exploited for industrial use of strains belonging to this species.

## 1. Introduction

Lactic acid bacteria (LAB) are a heterogeneous group of strains from different genera defined as Gram positive with a low G + C content in its genome, microaerophilic, and synthesize lactic acid as the main or only end product of their carbohydrate metabolisms. LAB are non-spore forming, and many of them are generally recognized as safe (GRAS) by the U.S. Food and Drug Administration (Makarova et al., 2006). They have been traditionally used to produce various fermented foods from animal products (e.g., milk, meat, and fish) or plants (e.g., vegetables, wine, and olives) (Zarour et al., 2017b). In fact, these so-called “starter strains” have the capacity to contribute to food safety and/or to offer one or more organoleptic, technological, nutritional, or health benefits through the synthesis of various products such as vitamins (e.g., folate and riboflavin) (Mosso et al., 2018; Mohedano et al., 2019), enzymes (e.g., phytase and lipases) (Anastasio et al., 2010; Zarour et al., 2018), antimicrobial peptides (e.g., bacteriocins) (Maldonado-Barragan and West, 2020) and exopolysaccharides (EPS) (Oleksy and Klewicka, 2016). The latter are polymers synthesized by LAB that differ in their chemical composition, type of linkage, degree and type of branching, and molecular mass. They are classified, according to their chemical composition, as follows: (1) hetepolysaccharide (HePS), which contain repeating units of several different types of monosaccharides, and (2) homopolysaccharides (HoPS), made of only glucose, fructose, or galactose residues (Pérez-Ramos et al., 2015). The use of the food-grade LAB EPS is currently of growing attention and commercial interest, particularly regarding dextran-type HoPS. Dextrans are α-glucan polymers, with major chains formed by glucoses joined by α-(1-6) linkages and with branches that arise from α-(1-2), α-(1-3), and α-(1-4) bonds (Monsan et al., 2001). These molecules are synthesized by several species of the genera *Leuconostoc* (Zarour et al., 2017a), *Lactobacillus* (Nácher-Vázquez et al., 2017), *Streptococcus* (Germaine and Schachtele, 1976), and *Weissella* (Malang et al., 2015; Llamas-Arriba et al., 2021) due to the catalytic activity of a single extracellular enzyme: the dextransucrase (Dsr). The Dsr enzymes are encoded by the *dsr* genes and are responsible for the synthesis of dextran catalyzing the hydrolysis of sucrose coupled to the transfer of glucose to the nascent α-glucan polymer and generating also free fructose (Werning et al., 2012). The molecular weight of dextrans varies from one polymer to another. There are high-molecular-weight dextrans that can reach up to 10^8^ Da (Besrour-Aouam et al., 2021) and others of low-molecular-weight not exceeding around 3 × 10^4^ Da (Claverie et al., 2017). Dextran application largely depends on their size. Low-molecular-weight dextrans are generally used for clinical application (Patel and Goyal, 2011), whereas high-molecular-weight ones have been the subject of several research studies aimed at stabilizing their potential as biothickeners, which could be synthesized *in situ* by bacteria, particularly from *Leuconostoc* and *Weissella* genus, during the fermentation of food matrices (Katina et al., 2009; Galle et al., 2012; Kothari et al., 2014). Moreover, according to recent studies, dextrans are postbiotics and could bring health benefits due to their antiviral and immunomodulatory properties (Nácher-Vázquez et al., 2015; Zarour et al., 2017a) and, thus, contribute to the clean label formulation of industrial food. Therefore, dextran-producing LAB have been tested in fermented dairy products in order to increase viscosity and reduce syneresis and, in low-fat cheese, to enhance creaminess (Kothari et al., 2014). Dextran-producing *Leuconostoc lactis* and *Weissella confusa* strains have been used as starters in pureed carrots to replace hydrocolloid additive and confer a suitable thick texture (Juvonen et al., 2015). These bacteria have also shown a special interest in the bakery industry since they contributed to the nutritional enrichment, shelf life and volume improvement of bread (Katina et al., 2009; Hu and Ganzle, 2018), and gluten-free bread (Galle et al., 2012; Rühmkorf et al., 2012).

The ecological role of dextrans for bacteria is not clearly understood. However, it has been suggested that they could play the role of carbon source as has been studied for *Streptococcus mutans*. In addition, to produce Dsr, this bacterium can produce an extracellular dextranase, which would produce isomaltooligosaccharides from dextran hydrolysis and these are ingested by bacteria and then converted into glucose by an intracellular glucan α-1,6-glucosidase (Klahan et al., 2018). Dextran could also be synthesized in response to environmental stress undergone by the bacteria. Thus, exposure to cold temperature (0–6°C) for a few weeks resulted in increased production of dextran by *Leuconostoc gelidum* and *Leuconostoc gasicomitatum* (Lyhs et al., 2004). Furthermore, upon cold shift during sourdough fermentation, *Weissella cibaria* 10 M was able to produce high dextran yield (Hu and Ganzle, 2018). This dextran production in response to cold temperature could be interpreted as a potential protective barrier for the bacteria, which would be an interesting property for a strain, given that recently studies have increasingly focused on the search for starters strains of interest not only of functional properties but also in terms of robustness and industrial performance with optimal survival during frozen storage, low-temperature fermentation during cheese ripening, or refrigerated storage of fermented products.

We have previously demonstrated that *Lc. lactis* AV1n isolated from a Tunisian avocado synthesizes a dextran-type HoPS with a main chain of glucopyranose units with α-(1-6) linkages and 9% substitution, at positions *O*-3, by side chains composed of a single residue of glucose and with a molecular mass of 2.61 × 10^8^ Da (Besrour-Aouam et al., 2019). This dextran is synthesized by a Dsr (named DsrLL), and the enzyme was visualized *in situ* by a zymogram (Besrour-Aouam et al., 2021). Moreover, the *dsrLL* gene of *Lc. lactis* AV1n has been previously sequenced and its homologous and heterologous overexpression confirmed that, indeed, the DsrLL enzyme synthesizes *in vivo* dextran (Besrour-Aouam et al., 2019). Furthermore, we have shown that expression of the *dsrLL* gene is induced in the presence of sucrose at low temperatures (Besrour-Aouam et al., 2019).

A better understanding of the responses to low temperatures may contribute to the optimization of fermented processes, storage of the products, and conservation conditions. Therefore, to further analyze DsrLL expression in response to low temperature and better understand the resistance mechanisms induced by the bacterium, a proteomic study of *Lc. lactis* AV1n in the presence of sucrose at 20°C and 37°C was carried out in this study. The obtained results, coupled with an analysis of the bacteria draft genome (Besrour-Aouam et al., 2021) and a predictive study of the folding of its *dsrLL* mRNA, have allowed us to present a road map of some of the regulatory mechanisms involved in the adaptation of this bacterium to cold temperatures.

## 2. Materials and methods

### 2.1. Bacterial strain and growth medium

The bacteria used in this study were *Lc. lactis* AV1n isolated from a Tunisian avocado and the recombinant *Lc. lactis* AV1n[pRCR21] strain (Besrour-Aouam et al., 2019). The latter harbors the pRCR21 plasmid, which carries the P*_*dsrLL*–*mrfp*_* transcriptional fusion encoding the mCherry protein under the control of the promoter of the *dsrLL* gene of *Lc. lactis* AV1n. Also, pRCR21 carries the *cat* gene encoding the chloramphenicol acetyl transferase, which confers Cm*^R^* to the bacterium. The bacteria were grown in MRS broth (Condalab, Torrejon de Ardoz, Madrid, Spain) supplemented with 2% glucose (MRSG) or with 2% sucrose (MRSS). In addition, the MRSS was supplemented with chloramphenicol (Cm) at 10 μg/ml, when *Lc. lactis* AV1n[pRCR21] was grown.

### 2.2. Analysis of bacterial growth and EPS production

Precultures of AV1n and AV1n[pRCR21] were generated by growth of the strains up to an optical density at 600 nm (OD_600 nm_) of 1.0 at 20°C, 30°C, or 37°C in MRSS. Then, cells from each culture were sedimented by centrifugation (9,300 × *g*, 10 min, 4°C), resuspended independently in fresh MRSS, and diluted 1:10 in MRSS. The cultures were then grown at 20°C, 30°C, or 37°C, respectively, as their corresponding precultures. The growth rate (μ) of the bacteria during the exponential phase was determined as previously described (Llamas-Arriba et al., 2021).

To determine the EPS concentration, samples were taken, when the cultures reached an OD_600 nm_ of 1.0 for the proteomic analysis experiments, or as indicated in the “2.9 Cold shock experiment” section and subjected to centrifugation (9,300 × g, 10 min, 4°C). Then, the dextran present in the culture supernatants was recovered by precipitation with three volumes of absolute ethanol and two washes with 80% (v/v) ethanol. Afterward, the polymer concentration was measured as neutral carbohydrate content as determined by the phenol–sulfuric acid method using a glucose calibration curve (Dubois et al., 1956). Experiments were performed in triplicate.

### 2.3. Cultures for preparation of total protein extracts

For the proteomic analysis of *Lc. lactis* cold response, the AV1n[pRCR21] strain was grown in MRSS medium at 20°C or 37°C. Precultures and cultures were generated as described in the “2.1 Bacterial strain and growth medium” section. The cultures were grown until they reached the point of transition between the end of the exponential phase and the beginning of the stationary phase. Then, a volume of bacterial culture containing 4.5 × 10^9^ colony-forming units (cfu) was harvested by centrifugation (11,269 × g, 10 min, 4°C), washed with cold PBS (pH 7.4), and stored at −80°C prior to protein extraction. Three cultures grown in either condition were prepared and stored.

### 2.4. Proteins extraction and quantification

For the protein extraction from the 6 cultures, the bacterial pellets stored at −80°C were thawed at 4°C and each was resuspended in 291 μl of cold PBS (137 mM NaCl, 2.7 mM KCl, 8 mM Na_2_HPO_4_, and 2 mM KH_2_PO_4_) pH 7.0 and 9 μl of 10% SDS. Then, each suspension was transferred into a Lysing Matrix B cryotube containing glass beads (MP Bio Germany GmbH) previously cooled to 0°C and subjected to two cycles of 45 s at a speed of 6, to break the bacterial cells in Ribolyser^®^ equipment (Hybaid). The tubes were then centrifuged (11,269 × g, 20 min, 4°C), and the upper suspensions containing the total protein extracts were collected and stored at −80°C. Protein quantification was performed using the Qubit^®^ Protein Assay Kit and the Qubit^®^ 1.0 fluorometer.

### 2.5. Sample preparation for proteomic analysis

For the analysis of the proteomic profile at 20°C or 37°C, protein extracts were fractionated by SDS-PAGE (in a 10% SDS-polyacrylamide gel) till the whole proteome had penetrated in the resolving gel (about 1 cm of total migration). Gels were stained with Colloidal Blue Staining Kit (Invitrogen). Each proteome was excised and cut into small pieces prior to manual in-gel digestion with trypsin. Excised bands were separately detained with 50 mM ammonium bicarbonate (ABC) (Sigma-Aldrich) and 50% acetonitrile (ACN) (Fisher Chemical). Samples were then reduced with 10 mM dithiothreitol (Bio-Rad) in 50 mM ABC and alkylated with 55 mM iodoacetamide (GE Healthcare Life Sciences) in 50 mM ABC. Then, gel pieces were digested with porcine trypsin (Thermo Fisher Scientific), at a final concentration 12.5 ng/ml in 50 mM ABC, overnight at 37°C. Peptides were extracted using 100% ACN and 0.5% trifluoroacetic acid (Sigma-Aldrich), purified using a Zip Tip (Millipore, Sigma-Aldrich), and dried. Finally, samples were reconstituted in 12 μl of 0.1% formic acid in water (Fisher Chemical) and the peptides were quantified using the Qubit^®^ Protein Assay Kit and the Qubit^®^ 1.0 fluorometer.

### 2.6. Mass spectrometry

Peptide separations were carried out on an Easy-nLC 1000 nano system (Thermo Fisher Scientific). For each analysis, the sample was loaded into an Acclaim PepMap 100 precolumn (Thermo Fisher Scientific) and eluted through rapid-separation liquid chromatography (RSLC) PepMap C18 column (Thermo Scientific) (50 cm long, 75 μm inner diameter, 2 μm particle size). The mobile phase flow rate was 300 nl/min using 0.1% formic acid in water (solvent A) and 0.1% formic acid and 100% acetonitrile (solvent B). The gradient profile was set as follows: 5–35% solvent B for 100 min, 35–45% solvent B for 20 min, 45–100% solvent B for 5 min, and 100% solvent B for 15 min. Four microliters of sample were injected. MS analysis was performed using a Q-Exactive mass spectrometer (Thermo Scientific). For ionization, 1,900 V of liquid junction voltage and 300°C capillary temperature were used. The full-scan method employed an m/z 400–1,500 mass selection, an Orbitrap resolution of 70,000 (at m/z 200), a target automatic gain control (AGC) value of 3e6, and maximum injection times of 100 ms. After the survey scan, the 15 most intense precursor ions were selected for MS/MS fragmentation. Fragmentation was performed with a normalized collision energy of 27 eV, and MS/MS scans were acquired with a starting mass of m/z 200, AGC target was 2e5, resolution of 17,500 (at m/z 200), intensity threshold of 8e4, isolation window of 2.0 m/z units, and maximum IT was 100 ms. Charge state screening was enabled to reject unassigned, singly charged, and equal or more than seven protonated ions. A dynamic exclusion time of 20 s was used to discriminate against previously selected ions.

### 2.7. Proteomic data analysis and accessibility

Raw files were processed with MaxQuant software, version 1.6.5.0 (Cox and Mann, 2008) using standardized protocols (Tyanova et al., 2016). Mass spectra *.raw files were searched against the *Lc. lactis* (UniProtKB/Swiss—Prot/TrEMBL, July 2019, 6,259 sequences) protein database, supplemented with the mCherry protein sequence. In the search parameters, the methionine oxidation was established as variable modification and carbamidomethylation of cysteines as a fixed modification. The minimal peptide length was set to 7 amino acids and a maximum of two tryptic missed cleavages were allowed. The results were filtered at 0.01 false discovery rate (FDR; peptide and protein level) and subsequently the MaxQuant output file (proteinGroup.txt) was loaded in the Perseus software, version 1.6.5.0 (Tyanova et al., 2016) for further statistical analysis. A minimum of 66% label-free quantification valid values per group was required for quantification. Missing values were imputed from the observed normal distribution of intensities for proteins expressed differentially. Then, a two-sample Student’s *t*-test with a permutation-based FDR was performed. Only proteins with an adjusted *p*-value ≤ 0.05, FDR 5%, and log2 ratio of ≥ 1.5 or ≤ 0.67 were considered as deregulated following the same criteria as in other proteomic analyses (Peláez-García et al., 2015; Liu et al., 2017; Romero-Gavilán et al., 2022; Solis-Fernández et al., 2022). The protein identification by nLC-MS/MS was carried out in the Proteomics and Genomics Facility at the Biological Research Center Margarita Salas (CIB-CSIC).

Data sets have been submitted to the PRIDE proteomics identification database (Pérez-Riverol et al., 2019, 2022; Deutsch et al., 2020) and are accessible under accession no. (AC) PXD037250.

Functional classification of detected proteins was performed using the Uniprot database^1^ and the Kyoto encyclopedia genes and genomes (KEGG) pathways database.^2^

### 2.8. Informatic analysis of predicted mRNA nucleotide sequence

Secondary structure predictions of the *dsrLL* and the *mrfp* transcripts were obtained by using the RNAfold web server (The ViennaRNA Web Services, version 2.4.18).

### 2.9. Cold shock experiment

For the analysis of the cold shock effect on strain survival in the presence or absence of dextran synthesis, *Lc. lactis* AV1n[pRCR21] was grown in MRSS or MRSG up to an OD_600 nm_ of 1.0 at 37°C. Then, the cells were sedimented by centrifugation (9,300 × g, 10 min, 4°C), resuspended in fresh MRSS or MRSG, diluted 1:10 in MRSS or MRSG, respectively, and grown at 37°C till the end of the exponential phase. The cells were then harvested by centrifugation at room temperature (9,300 × g, 3 min at 21°C), resuspended at 37°C in prewarmed MRSS or MRSG medium, respectively, and immediately chilled to 10°C (cold shocked) and maintained at this temperature for 18 h. Afterward, 1 ml of each culture was frozen at −80°C without any cryoprotective agent and maintained frozen for 3 days prior to the evaluation of cell survival after thawing.

The fluorescence of the mCherry protein in the bacterial cultures, before and after the exposure to 10°C, was measured by excitation at a wavelength of 587 nm and detection of emission at 610 nm using a Varioskan Flask System (Thermo Fisher Scientific), as previously described (Besrour-Aouam et al., 2019). Colony-forming units/ml were determined prior to and after the cold shock at 10°C and after thawing, frozen-cold pretreated cultures by serially diluting in saline solution (NaCl 0.85%), spread plating, and incubation overnight.

EPS concentration in supernatants of cultures subjected to the 10°C cold shocks was determined as described in the “2.1 Bacterial strain and growth medium” section. The experiments were performed in triplicate and mean values, with standard deviations, are presented.

### 2.10. Statistical analysis

All experiments were performed in triplicate. The results are expressed as means, with the corresponding standard deviations. For the analysis of mCherry fluorescence and survival of *Lc. lactis* AV1n[pRCR21] under cold shock, differences before and after treatment as well as between influence of carbon source were analyzed. The results were subjected to a one-way analysis of variance (ANOVA). A two factorial randomized complete block design with interactions was performed, with experiments on different days treated as random blocks. A *p*-value of ≤ 0.05 was considered significant. For the analysis of survival of strains under cold shock, a mean pairwise comparison of all conditions tested by a Tukey’s test (α = 0.05) was performed. All analyses were done with the R software, version 4.2.1 (R Core Team, 2022).

## 3. Results and discussion

### 3.1. Selection and generation of cultures for protein analysis

Our previous results indicated that *Lc. lactis* AV1n DsrLL, which synthetized dextran using sucrose as substrate, was involved in the response of the strain to low-temperature stress since this bacterium was able to produce higher levels of dextran at low temperature (Besrour-Aouam et al., 2019). Furthermore, we have shown that a transcriptional activation of the *dsrLL* gene expression in *Lc. lactis* takes place when the temperature decreases (Besrour-Aouam et al., 2019). This conclusion was inferred from the analysis of expression of the P*_*dsrLL*_*_–_*_*mrfp*_* transcriptional fusion in *Lc. lactis* AV1n[pRCR21] by measuring the fluorescence of the mCherry protein, the product of the *mrfp* gene at different temperatures.

**FIGURE 1:**
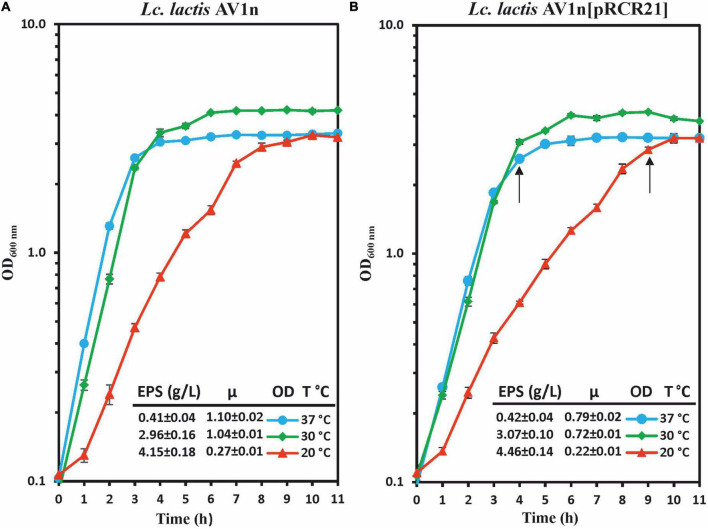
Analysis of growth and dextran production by *Lc. lactis* AV1n **(A)** and *Lc. lactis* AV1n[pRCR21] **(B)**. Bacteria were grown in MRSS at 20°C, 30°C, and 37°C. Levels of dextran produced by the LAB at OD_600 nm_ = 1.0 are also depicted. The mean values and the standard deviation of three independent experiments are depicted. Arrows indicated the time at which samples for the proteomic analysis were taken.

Therefore, a proteomic analysis of the *Lc. lactis* AV1n[pRCR21] at different temperatures could provide information on alterations of the DsrLL levels encoded by the *dsrLL* gene present in the chromosome and, in addition, could estimate transcriptional activation of *dsrLL* gene expression by measurement of the mCherry levels encoded by the P*_*dsrLL*_*_–_*_*mrfp*_* carried by the pRCR21 plasmid. However, prior to performing the proteomic study, it was assessed a comparative analysis of growth and EPS production in the presence of sucrose by the parental AV1n and the recombinant AV1n[pRCR21] strains at 20°C, 30°C, and 37°C (Figure 1). Concerning the EPS production (measured when the cultures reached an OD_600 nm_ = 1.0), almost the same levels were produced by the two strains at each of the three temperatures tested (4.1–4.5 g/L at 20°C, 3 g/L at 30°C, and 0.4 g/L at 37°C). Moreover, as previously observed (Besrour-Aouam et al., 2019), both strains were able to produce 10-fold higher levels of dextran at 20°C than at 37°C (Besrour-Aouam et al., 2019). Concerning to the growth, both bacteria showed the same pattern at the three temperatures tested. At 20°C, an optimal growth with the lowest μ was observed [μ = 0.27 h^–1^ or 0.22 h^–1^ for AV1n or AV1n(pRCR21) strains, respectively], which seems to stimulate a self-adjustment for the bacteria to develop cold resistance mechanisms. Moreover, at 30°C and 37°C, for each strain, the growth rate was similar at both temperatures, but it was lower for the recombinant strain than for the parental strain (0.7 and 0.8 h^–1^ vs. 1.0 and 1.1 h^–1^). However, a marked decrease in growth was not observed for *Lc. lactis* AV1n[pRCR21].

To gain insight into the response of *Lc. lactis* to low temperature, the analysis of the protein profile of *Lc. lactis* AV1n[pRCR21] was carried out in the presence of sucrose at 20°C and 37°C. Moreover, taking in consideration growth differences at these two temperatures, samples for preparations of total protein extracts were withdrawn when the cultures were in the same growth state, the transition from exponential to stationary phase (at OD_600 nm_ = 2.6 or OD_600 nm_ = 2.8, after 4 or 9 h of growth at 37°C or 20°C, respectively; Figure 1).

### 3.2. Functional analysis of the differentially expressed proteins

Previous analysis of the amino acid sequence of the DsrLL supported its extracellular location and revealed that the protein contains an LPXTG cell wall anchor domain at its carboxyl-terminal region. Thus, the overall analysis predicted that the *dsrLL* gene encodes an active DsrLL enzyme bound to the cell wall (Besrour-Aouam et al., 2019). Thus, the proteomic study was carried out by nanoHPLC-MS-MS analysis of cellular protein extracts after tryptic digestion. Comparison with the *Lc*. *lactis* database (Uniprot-TrEMBL) identified 1,385 proteins, of which 337 were differentially expressed. Two hundred and four were overproduced at 20°C and 133 were downregulated in comparison with the levels detected at 37°C. Only proteins with an adjusted *p*-value ≤ 0.05, FDR 5%, and log2 ratio of ≥ 1.5 or ≤ 0.67 were considered differentially expressed (Supplementary Table 1). Moreover, the increase in proteins levels varied as follows: (1) at 20°C, from 53-fold for the phosphonate ABC transporter substrate-binding protein to 1.5-fold for the UDP-galactopyranose mutase, and (2) at 37°C, from 17-fold for an uncharacterized protein (Ac: A0A1B1ZZ24) to 1.4-fold for the type I restriction enzyme R Protein.

In addition, 19 proteins were only detected at 20°C (Supplementary Table 2) and 9 proteins were only identified at 37°C (Supplementary Table 3). The first group included the DsrLL (Ac: A0A411G111) and a CspA cold shock protein (Ac: A0A1B2A2G5), and the second group included the ClpE protease and the DNA repair protein Rec O.

**FIGURE 2:**
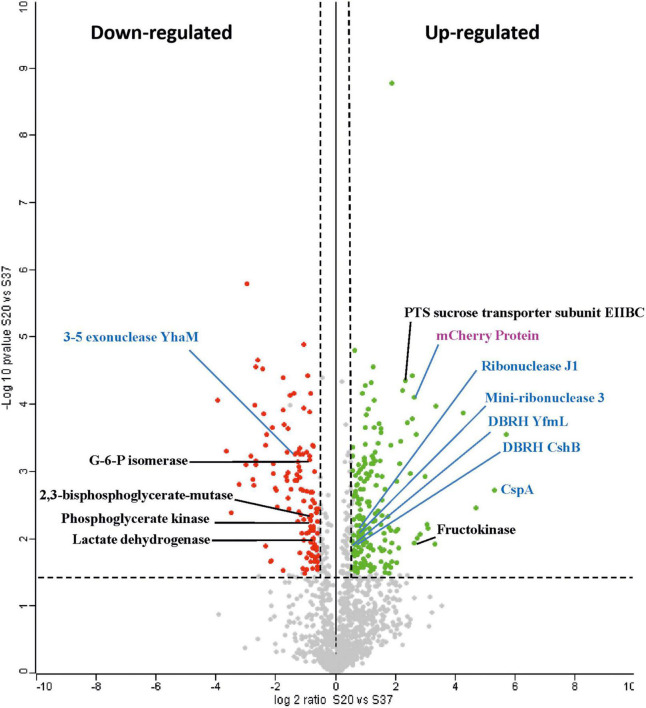
Volcano plot of upregulated and downregulated proteins in *Lc. lactis* AV1n[pRCR21] cultures grown to 20°C vs. 37°C. A test comparison of 1,385 quantified proteins was performed. Only proteins with a differential expression level in the range ± 10 are shown (Supplementary Table 1). The *x*-axis is log2 ratio of protein expression differences between two groups (S20°C vs. S37°C), and the *y*-axis is the *p-*value based on −log10. Red circles indicate differentially expressed proteins (downregulated), and green circles indicate differentially expressed (upregulated). Gray dots indicate non-differentially expressed proteins. The dashed horizontal line indicates the threshold of the adjusted *p*-value ≤ 0.05, FDR 5%, whereas dashed vertical lines enclose an area with a log2 ratio ± 0.58 (i.e., ratio ≥ 1.5 or ≤ 0.67). Blue color is used to group proteins involved in cold shock response, whereas black color is for those involved in carbohydrate metabolism. The mCherry protein is denoted in purple color.

Figure 2 depicts a volcano plot of the distribution of the total proteins quantified with the upregulated at 20°C in green and the downregulated at 20°C in red. Among the upregulated proteins, only 28 showed a log2 ratio > 2, including the mCherry, and of the downregulated proteins, only 33 had a log2 ratio < −2, including the universal stress protein UspA, indicating that both 20°C and 37°C have a stressful effect on *Lc. lactis* AV1n.

**FIGURE 3:**
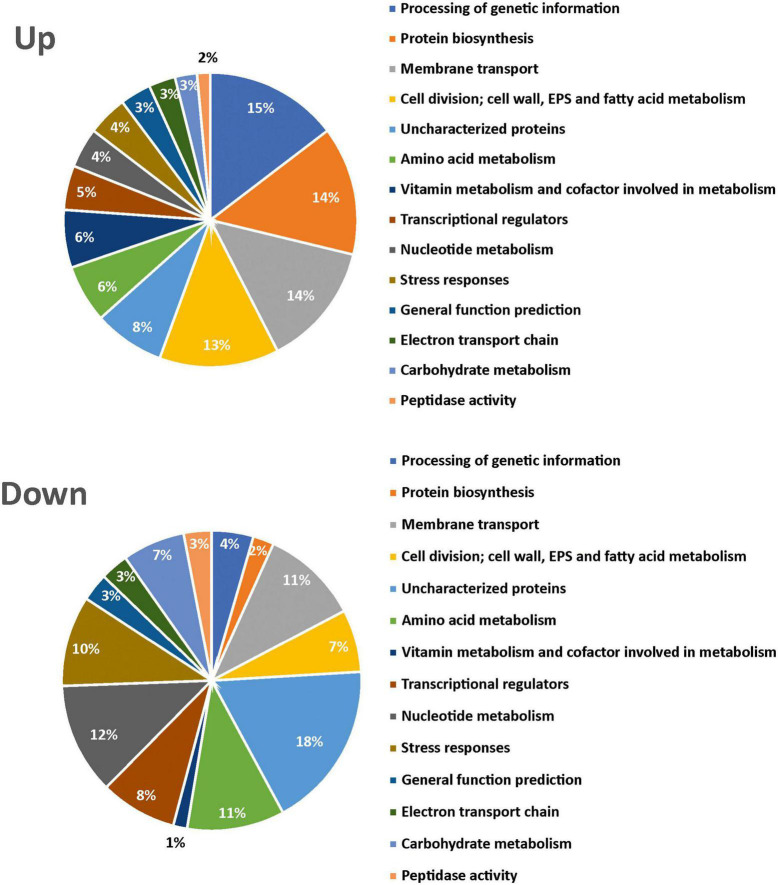
Pie chart representation in *Lc. lactis* AV1n[pRCR21] of upregulated and downregulated proteins at 20°C vs. 37°C. Fourteen different categories and their relative percentage are depicted.

In addition, a functional classification of differentially expressed proteins and determination of the biological pathway to which they belong were performed using the Uniprot database and the KEGG pathway database, respectively. The proteins were included in 14 categories, and the proportion of each one is shown in Figure 3. Proteins overexpressed at 20°C were mainly involved in genetic information processing (15%), protein biosynthesis (translation 14%), components of transport (14%), and cell division, cell wall, EPS, and fatty acids metabolism (13%), with DsrLL being included in this last category, as expected from our previous results (Supplementary Tables 1, 2). Proteins underexpressed at 20°C, apart from hypothetical proteins, were mainly involved in nucleotide (12%), carbohydrate (7%), and amino acid (11%) metabolism (Supplementary Tables 1, 3). The 19 proteins only detected at 20°C belonged to 8 categories (EPS biosynthesis, stress response, processing of genetic information, membrane transport, transcriptional regulators, general functions, protein biosynthesis, or uncharacterized) (Supplementary Table 2). Furthermore, the 9 proteins, only detected at 37°C, belonged to eight different categories (stress response, processing of genetic information, amino acid, carbohydrate or fatty acid metabolisms, cofactors of metabolism, electron transport chain, or uncharacterized) (Supplementary Table 3).

### 3.3. Analysis of key proteins involved in the regulation of the *dsrLL* expression transcript

In response to a temperature decrease, microorganisms adapt by triggering a physiological response, which includes the synthesis of cold shock proteins (Csps). 4 Csps encoding genes have been detected after the analysis of the *Lc. lactis* AV1n draft genome (Besrour-Aouam et al., 2021). Also, in this genome, we have detected 3 DEAD box RNA helicases (DBRH) coding genes, showing that this bacterium is genetically prepared to counteract cold stress. Consistent with this observation, one CspA was only detected at 20°C (Ac: A0A1B2A2G5), and a 40-fold upregulation of other CspA (A0A0N8VZ44), as well as a moderate upregulation of 3 DBRH (3.7-, 1.8-, and 1.5-fold upregulation of CshA, CshB, and YfmL, respectively), was observed at this temperature (Supplementary Table 1).

Furthermore, higher levels of GrpE (1.6-fold upregulation), DnaK chaperone (3.4-fold upregulation), and UspA universal stress protein (3.8-fold upregulation) were observed at 37°C (Supplementary Table 1). These proteins are implicated in heat shock response and their induction suggested that this temperature may impose a moderate heat stress for this bacterium (Champomier-Vergès et al., 2010; Rossi et al., 2016).

DsrLL was only detected at 20°C, showing that low levels of Dsr catalyzing the synthesis of 0.4 g/L of dextran at 37°C were undetectable by the proteomic analysis performed here (Supplementary Table 2). Furthermore, these results confirmed, as expected, that a substantial upregulation of DsrLL levels takes place at 20°C, indicating that this enzyme plays a prominent role in the adaptation of *Lc. lactis* AV1n to low temperature.

The high levels of DsrLL detected at 20°C also correlate with our previous observation of a significant activation of transcription from P*_*dsrLL*_* at low temperature (Besrour-Aouam et al., 2019). In addition, the proteomic analysis revealed a 6.2-fold increase of the mCherry protein at 20°C vs. 37°C, confirming the induction of DsrLL production at the transcriptional level from the P*_*dsrLL*_* promoter. However, the increase in the mCherry levels at 20°C was lower than that of DsrLL (>50-fold), indicating that probably a more complex regulation of the *dsrLL* gene expression was taking place.

Cold-inducible promoters regulate the expression of genes coding for specific proteins that will ensure the survival of the bacterium. This is the case, for example, of the Csps proteins (Barria et al., 2013), which are found in LAB (Kim et al., 1998; Wouters et al., 1999). In addition to the regulation of the expression of genes encoding Csps, other cold-inducible genes seem to be controlled at several levels. It is thought to take place at the level of transcription, translation, and mRNA stability and to involve several characteristic genetic elements. The cold shock promoters generally include sequences rich in AT nucleotides (UP-element), located in the −65 region upstream of the transcription start site, which would stimulate gene transcription by interacting with the α subunit of the RNA polymerase at low temperature (Wouters et al., 2000; Singh et al., 2014). These promoters also possess an untranslated region (UTR) of approximately 100 nt between the promoter sequence and the ribosomal binding site (SD sequence) capable of modulating translation through the formation of secondary structures and mRNA–protein interactions (Singh et al., 2014).

**FIGURE 4:**
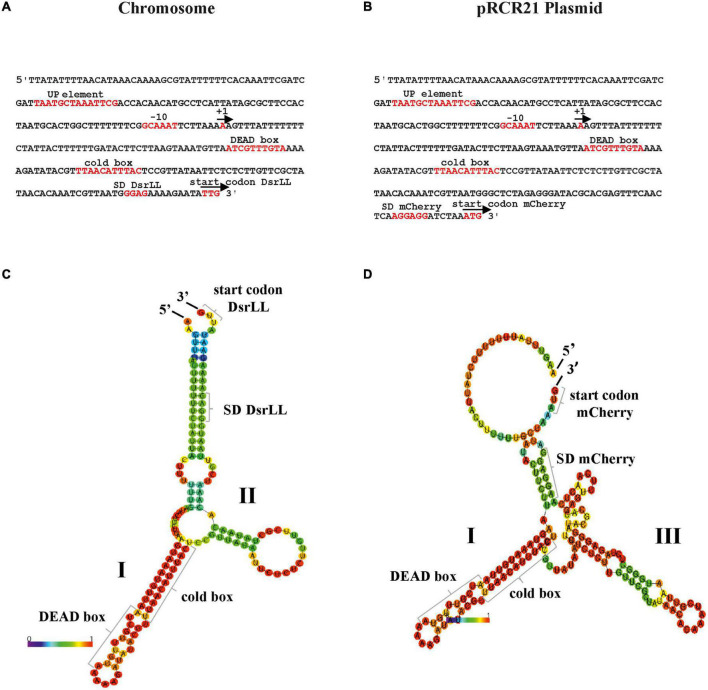
Regulatory signals for expression from P*_*dsrLL*_* in *Lc. lactis* AV1n[pRCR21]. The P*_*dsrLL*_* promoters of *Lc. lactis* AV1n and putative regulatory elements involved in their strength when present in the bacterial chromosome **(A)** or the pRCR21 plasmid **(B)** are shown. Also, the predictive folding of the UTR of the *dsrLL* **(C)** and *mrfp* **(D)** transcripts is depicted. In **(A,B)**, the + 1 transcription initiation site, −10 region, UP-element, cold box, DEAD box, as well as the Shine–Dalgarno (SD) sequences and the translation initiation sites of *dsrLL* **(C)** and *mrfp* **(D)** genes are framed in red. **(C,D)** Indicate the location and sequence of the 5′- and 3′-ends, secondary structures I and II in **(C)** and structures I and III in **(D)**, DEAD box, cold box, ribosomal binding sites, and translation initiation codon (start codon) of the d*srLL* **(C)** or *mrfp* **(D)** genes. The folding of the UTR from the chromosome and pRCR21 plasmid has a Δ*G* of −29.78 and −39.99 kcal/mol, respectively.

We have previously detected the location of the start site of the *Lc. lactis* AV1n *dsrLL* transcript by primer extension (Besrour-Aouam et al., 2019). This allowed us to predict the −10 region of the P*_*dsrLL*_* promoter and to determine the extent of its UTR in the bacterial chromosome (Besrour-Aouam et al., 2019; Figure 4). In the case of *Lc. lactis* AV1n[pRCR21], the P*_*dsrLL*_* is present in two copies, one in the chromosome (Figure 4A) and the other in the pRCR21 plasmid (Figure 4B). Both promoters contain the same AT-rich sequence “TAaTGCTaaAttcg” similar to the consensus sequence of the “UP-element” (TACTNCTGGAAAGT) located upstream of the −10 region (Figures 4A, B). A UTR is also present, which will not be translated, of 141 nucleotides in the chromosome and of 173 nucleotides in the plasmid, which both contain potential low-temperature regulation-related sequences, such as the cold box “TtAACAttTaC” close to the consensus sequence (TGAACAACTGC) and DEAD box “AtCgtTtGTA” very similar to the consensus sequence (AACAGTGGTA) (Figure 4), which could act as a binding site for the regulatory proteins Csp and DBRH (Singh et al., 2014).

These proteins act as RNA chaperones and are essential for efficient protein synthesis and cell survival at low temperatures. Moreover, both types of proteins are known to be overproduced at low temperatures in many Gram-positive and Gram-negative bacteria (Iost et al., 2013) and may play a role in the upregulation of DsrLL expression at the post-transcriptional level. In fact, as stated earlier, the overproduction of the 2 CspA designated as the major cold shock proteins (Beales, 2004), with a high induction level, as well as 3 DBRH (CshA, CshB, and YfmL) (Supplementary Table 1) was detected at 20°C.

The mRNAs, being single-stranded nucleic acids, would tend to fold and form secondary structures that prevent the efficient initiation of translation. Predictive folding of the UTR of the *dsrLL* (Figure 4C) and *mrfp* (Figure 4D) mRNAs with the RNAfold program revealed in both regions the presence of a complex secondary structure I, which contains the cold and DEAD boxes, followed by a small secondary structure II in the former transcript and a more complex secondary structure III in the latter, accompanied by differential ribosomal binding sites blockings in a double-stranded region. Moreover, the total Δ*G* of the chromosomal and plasmidic UTR folding was different (−29.78 kcal/mol vs. −39.99 kcal/mol).

**FIGURE 5:**
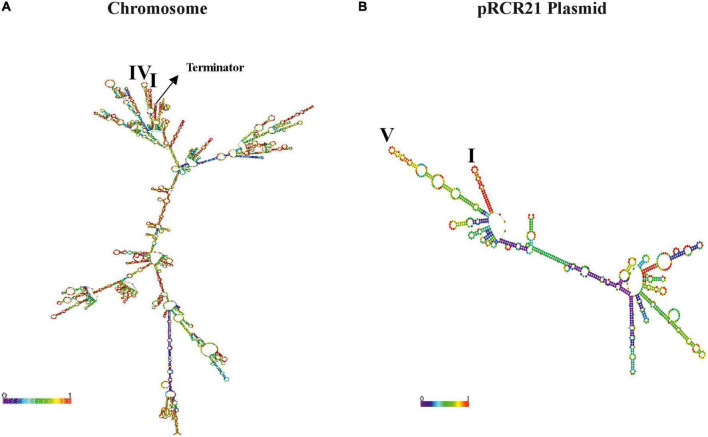
Predicted folding of the transcripts driven from P*_*dsrLL*_* promoter in *Lc. lactis* AV1n[pRCR21]. **(A)** The chromosomal *drLL* mRNA is depicted, along with the location of complex secondary structures I and IV as well as the Rho-independent terminator. **(B)** The *mrfp* transcript encoded by pRCR21 is depicted, along with the location of complex secondary structures I and V.

Furthermore, the prediction of the folding of the entire transcripts (Figure 5) revealed that structure I was still present in both the *dsrLL* (Figure 5A) and the *mrfp* (Figure 5B), whereas instead of structures II and III, there were now predicted structures IV and V. Therefore, due to these differences, and as expected for the nature of the transcriptional (non-translational) P*_*dsrLL*_*_–_*_*mrfp*_* fusion, its expression was predominantly valid only to assess the strength of the promoter at different temperatures and not for the analysis of post-transcriptional regulations.

**FIGURE 6:**
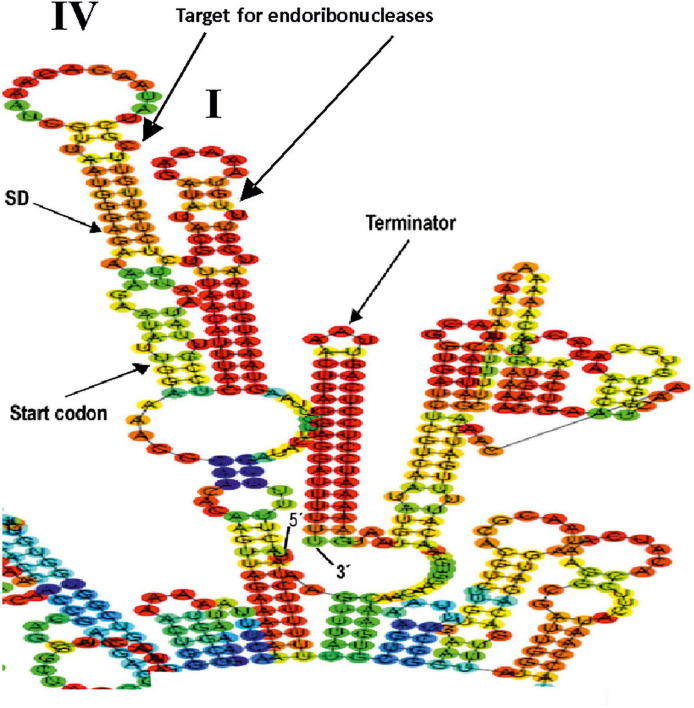
Details of predictive folding of the *dsrLL* transcript driven from P*_*dsrLL*_* promoter encoded by in *Lc. lactis* AV1n[pRCR21] chromosome. The 5′- and 3′-ends of the mRNA are indicated. The sequences of the secondary structures I and IV, ribosomal binding site (SD), *dsrLL* translation initiation codon (start codon), and Rho-independent terminator are shown in detail.

Concerning the potential post-transcriptional regulation of *dsrLL* gene expression in *Lc. lactis* AV1n, a detailed analysis of the 5′ and 3′ regions (enlarged in Figure 6) of the total transcript indicates that the complex structures I (carrying the CspA and DBRH proteins-binding boxes) and IV (carrying the ribosomal binding *SD* sequence and the DsrLL translational start codon) could be the target for endonucleases. Also, the existence of a transcriptional ρ independent terminator at the 3′-end of the *dsrLL* mRNA, which will not only be involved in the dissociation of RNA polymerase during transcription but could also partially prevent mRNA degradation by 3′–5′ exonucleases. In fact, among these exonucleases, the enzyme YhaM was detected as negatively expressed (2.3-fold downregulated) in *Lc lactis* AV1n at 20°C and has been previously studied in *Streptococcus pyogenes* (Broglia et al., 2020).

On these bases, the stabilization of the secondary structures at low temperatures can occur and folding of the UTR of the *dsrLL* mRNA could slow down the transcription performed by the RNA polymerase at the complex secondary structure. However, the binding to the DEAD and cold boxes of the abundant DBRH and CspA proteins at 20°C should prevent the formation of the secondary structure I. In fact, these 2 types of proteins may have a cooperative activity during cold stress response as reported previously in *Bacillus subtilis* (Hunger et al., 2005). The CshA exhibited the highest fold-change among the 3 DBRH overexpressed at 20°C (Supplementary Table 1), and recent studies revealed that this protein was able to auto-regulate its own expression by binding to the UTR of its mRNA (Salze et al., 2020). In addition, proteins CshA and CshB could destabilize the unfavorable secondary structure I by unwinding them in an ATP-dependent manner, then the single-stranded mRNA can successively be bound by Csps to prevent refolding until translation is initiated at the ribosome. Furthermore, DBRH CshA seems to play a more important role in RNA processing since *Enterococcus faecalis* Δ*CshA* gene mutant was the most stress impacted strain (Salze et al., 2020).

The control of mRNA turnover is essential in bacteria to allow rapid adaptation to stress environments. It directly affects protein synthesis by modulating the amount of mRNA available for translation. The decay of the transcripts is usually initiated by endoribonucleases, which produce intermediate fragments that are subsequently degraded by exoribonucleases. Structures I and IV could be stabilized in the total mRNA folding and they could be the target of an endonuclease. The proteomic analysis revealed increased levels of two endonucleases at 20°C: the ribonuclease J1 (1.9-fold upregulated) and the mini-ribonuclease III (2.8-fold upregulated). Ribonuclease J1 was the first ribonuclease identified in Gram-positive bacteria to be able to perform both types of activity, endonucleolytic and 5′ exonucleolytic, using a single catalytic site (Laalami and Putzer, 2011), and it could be involved in mRNA decay. Mini-ribonuclease III belongs to the class of ribonucleases that bind to and cleave double-stranded RNAs. It was identified as an enzyme involved in the maturation of 23S ribosomal RNA in *B. subtilis* (Redko et al., 2008). In addition, mini-ribonuclease III from different bacterial species can cleave long double-stranded RNAs, such as mRNA complex secondary structure, with a certain degree of sequence specificity (Glow et al., 2015).

In summary, it appears that Csps, DBRH, and ribonuclease(s), commonly known as cold shock response gene families in bacteria, tightly regulate RNA metabolism to adapt to a changing environment and to optimally activate *dsrLL* gene translation.

**FIGURE 7:**
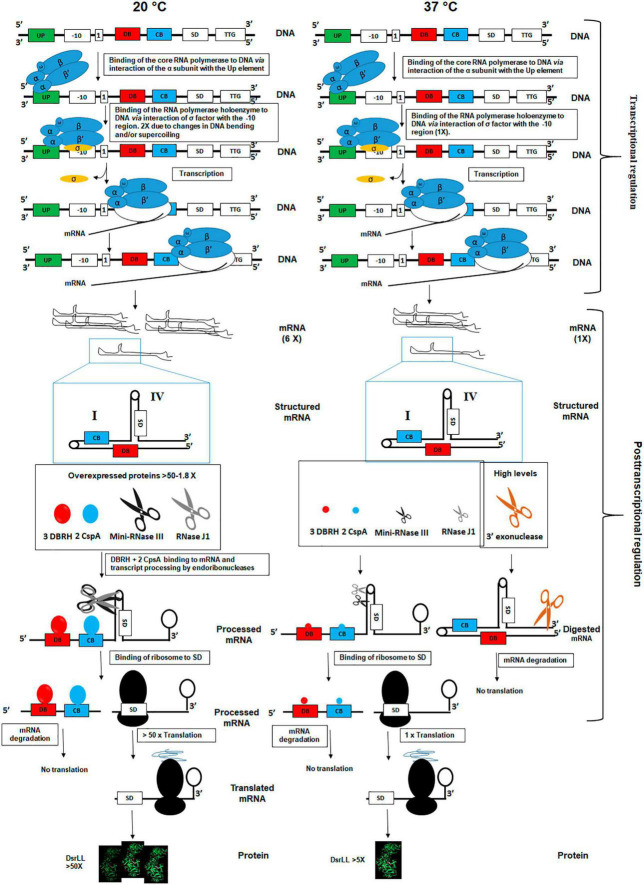
Model of transcriptional and post-transcriptional regulation of *dsrLL* gene expression in *Lc. lactis* at 20°C and 37°C. The influence of DNA bending and supercoiling at the two temperatures on the efficient utilization of promoter by RNA polymerase are indicated. Also, influence of mRNA folding vs. 3 DBRH and 2 CspA binding as well as ribonucleases binding and catalysis in the fate and turnover of the transcript at 20°C and 37°C are proposed.

Thus, based on the current knowledge, and the study performed here, a regulatory model for the expression of DsrLL at 20°C and 37°C is proposed (Figure 7). At the transcriptional level, it seems that temperature-dependent alterations of the DNA negative supercoiling (Wouters et al., 2000) and DNA bending (Prosseda et al., 2010) result in a more efficient binding of the α subunit of the RNA polymerase to the UP element and as a consequence of higher synthesis of the *dsrLL* mRNA at 20°C. Then, at both temperatures, only transcripts that interact with Csps and DBRH will not contain structure I and will be correctly processed at structure IV by an endoribonuclease (presumably by mini-RNase III enzyme and/or ribonuclease J1). If J1 will be involved in the processing of structure IV, after its endonucleolytic incision at the structure, its 5′ exonucleolytic activity could generate an RNA species, in which the SD sequence could be exposed for ribosomal binding and subsequent translation of DsrLL, contributing to mRNA turnover, a role previously proposed for this ribonuclease (Condon, 2010). According to the proteomic results obtained for the various proteins involved in stability, processing, and degradation of these molecules will be substantially more abundant at 20°C than at 37°C. Consequently, translation of this mRNA species will be most efficient at low temperature. In addition, mRNA molecules containing the secondary structure I will be more abundant at 37°C, due to the lower levels of Csps and DBRH proteins. Also, they could be processing by endoribonucleases and degraded by exonucleases. In this context, the higher abundance of the 3′–5′ exonuclease YhaM at 37°C should potentiate degradation of untranslated mRNA molecules.

Finally, another post-translational factor seems to influence the coupling of the Dsr with a cold response. This enzyme acts extracellularly, and after being synthesized in the bacterial cytosol, it is translocated through the cell membrane due to a signal peptide sequence in the N-terminal region of the protein (Besrour-Aouam et al., 2019), which is cleaved off post-translationally to lead the protein to the secretory pathway, by a signal peptidase (Nielsen et al., 1997; van Roosmalen et al., 2004). Therefore, it is probable that the high levels of DsrLL synthesized at 20°C will be efficiently secreted because it was detected at this temperature a 2.9-fold increase of the peptidase I, accompanied by a 1.7-fold upregulation of the protein translocase subunits SecA and SecY, members of the secretory pathway (Supplementary Table 1).

### 3.4. Effect of cold temperature in *L. lactis* AV1n carbohydrates metabolism and cell wall development

In addition to the increase in DsrLL production in response to the drop in temperature, the bacterium possesses other cold resistance mechanisms, including the activation of metabolic pathways. Indeed, membrane transporters (for active transport and secretion of molecules) are among the group of proteins most overexpressed at 20°C (Supplementary Tables 1, 2 and Figure 2) and include the PTS sucrose transporter subunit EIIBCA with a 5.9-fold increase at this temperature. This transporter ensures the internalization of sucrose, which was the only carbon source available in the medium in the proteomic study performed here.

**FIGURE 8:**
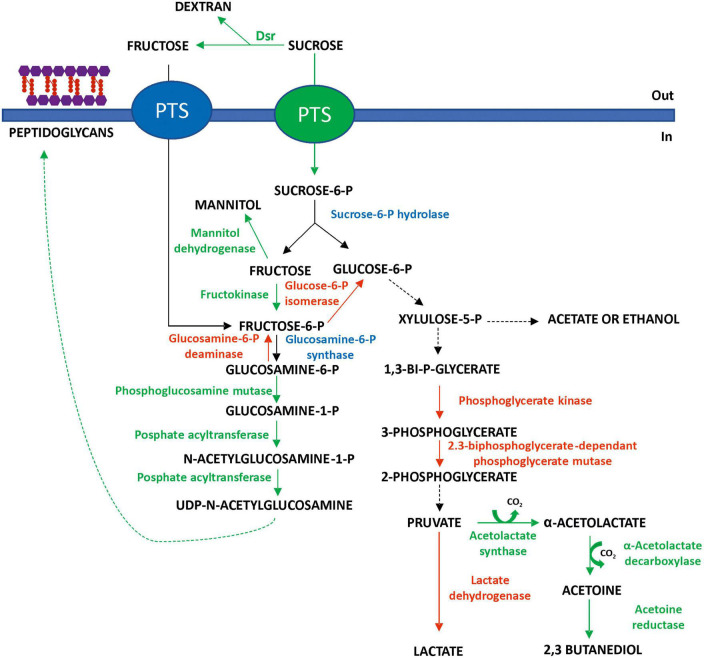
Prediction of major carbohydrate utilization pathways in *Lc. lactis* AV1n at 20°C in the presence of sucrose as the sole carbon source, suggested by proteomic analysis. Red indicates the downregulated enzymes, green indicates the upregulated ones, and blue indicates non-differentially expressed enzymes.

Moreover, the machinery for the transport and/or metabolism of other disaccharides or unrelated monosaccharides seems to be downregulated in the absence of the substrates (Supplementary Table 1). Thus, at 20°C, a 2.3-fold decrease in the maltose phosphorylase was detected. Moreover, the PTS mannose transporter subunits IID (Ac: A0A1B2A3C0) and EIIAB (A0A1B2A1F3) were 1.7- and 1.8-fold downregulated, respectively, as well as the mannose-6-phosphate isomerase, whose levels were decreased by 6.4-fold. Therefore, it seems that in *Lc. lactis* AV1n, at low temperature, certain metabolic pathways, rather than others, are favored to ensure its survival (Figure 8).

Thus, in addition to the hydrolysis of some sucrose molecules by DsrLL, at 20°C, other molecules of this disaccharide pool could be internalized and then hydrolyzed by a sucrose-6-P hydrolase into glucose-6-P and fructose, monosaccharides that will be directed toward different pathways. Among the transcriptional regulators, downregulated at 20°C, a 2.4-fold decrease in the sucrose operon repressor ScrR (Ac: A0A1B1ZZZ0) was detected, which precedes the mannitol dehydrogenase and the fructokinase genes in the *Lc. lactis* AV1n genome (Besrour-Aouam et al., 2021) and which seems to co-regulate them. It can be assumed that the decrease in the expression of this repressor would lead to a slight increase in the expression of mannitol dehydrogenase (not detected in the proteomic analysis) and thus in the production of mannitol, previously observed, when the bacterium was grown in the presence of sucrose (Besrour-Aouam et al., 2021). This implies that some of the fructose released during dextran synthesis and hydrolysis of internalized sucrose will be metabolized by this biosynthetic pathway (Figure 8). This probable increase in mannitol production triggered by the bacterium may be necessary for two reasons: (1) as an alternative pathway to regenerate NAD+ and (2) to stabilize lipid and protein structures of the cell membrane (Wisselink et al., 2002), a role that is consistent with the bacterial membrane integrity protection under environmental stress. Furthermore, the 1.8-fold decrease in glucose-6-P isomerase, detected at 20°C, implies that the fructose-6-P generated by fructose phosphorylation by the 6.2-fold upregulated fructokinase will be preferentially directed toward other pathways, in particular that of peptidoglycan synthesis (refer to details mentioned later), rather than glycolysis (Figure 8).

Moreover, the glucose-6-P glycolysis pathway should have been slowed down since a 1.5-fold under-regulation of the 2,3-biphosphoglycerate-dependant phosphoglycerate mutase was observed, which is present in two copies in the proteome (Ac: A0A0Q0U9Y5 and A0A1 × 0V3P9), as well as the 1.8-fold decrease in the phosphoglycerate kinase and the 1.5-fold downregulation of lactate dehydrogenase levels (Supplementary Table 1). These 3 enzymes are involved in the heterolactic fermentation pathway of the bacterium (Figure 8), and the detected reduction of the level of the enzymes, which should result in their lower catalysis, could contribute to the decrease in the bacterium growth rate observed at 20°C (Figure 1).

Furthermore, it seems that at 20°C, the pyruvate generated from glycolysis will be preferentially directed to the acetoin and 2,3-butanediol biosynthetic pathway (Xiao and Xu, 2007) since a 2.4-fold increase in the acetolactate synthase and the α-acetolactate decarboxylase levels was observed, as well as a 2.0-fold upregulation of acetoin reductase (Figure 8). This upregulation of the pathway will not only reduce acid production in the medium and thus limit acid stress but it will be also a mechanism that the bacterium has evolved to store energy. Furthermore, due to the reversible transformation between acetoin and 2,3-butanediol coupled with NAD^+^/NADH conversion, this pathway has been considered to participate in the regulation of the NAD^+^/NADH ratio in bacteria (Xiao and Xu, 2007).

In addition, despite the decrease in the growth rate of *Lc. lactis* AV1n[pRCR21], enzymes involved in the synthesis of peptidoglycans (Figure 8), a major component of the cell wall of Gram-negative and Gram-positive bacteria, were up-regulated at 20°C. In particular, a 1.7- and 19.5-fold increase in phosphoglucosamine mutase and phosphate acyltransferase, 2 enzymes involved in the synthesis of UDP-*N*-acetylglucosamine, which is one of the key elements in peptidoglycan biosynthesis. By contrast, enzymes associated with the recycling of these molecules to the glycolysis pathway such as *N*-acetylglucosamine-6-phosphate deacetylase and glucosamine-6-phosphate deaminase were downregulated (4.3- and 3.4-fold decrease, respectively), probably to favor the cell wall biosynthetic pathway (Figure 8). This behavior has been previously observed for *Lactiplantibacillus plantarum* K25, in which the biosynthesis of its cell wall components was increased following exposure to cold (Liu et al., 2020).

In addition to the increase in proteins involved in cell wall synthesis, in *Lc. lactis* AV1n at 20°C a 1.6-fold increase in DNDP-4-keto-6-deoxy-glucose 2,3 dehydratase levels was observed (Supplementary Table 1), an enzyme involved in the heteropolysaccharide formation pathway of the bacterial capsule. This can be explained by the fact that the cell wall is the first line of defense against external stresses, and this mechanism took place in *Lc. lactis* AV1n synchronically with an increase in DsrLL expression.

The production of dextran in *Lc. lactis* included in the response to cold exposure could contribute to the decrease in bacterial growth detected at 20°C (Figure 1). This behavior has also been reported for *W. cibaria* 10 M, which showed a higher growth rate at 30°C; while Dsr expression was higher at 15°C (Hu and Ganzle, 2018). Moreover, proteomic analysis of *Sphingopyxis alaskensis* showed that several proteins related to the cell wall, membrane, EPS biosynthesis, and envelope biogenesis had a higher abundance at 10°C (Ting et al., 2010). Therefore, the generation of a biofilm matrix composed of dextran and the reinforcement of the cell wall could constitute a physical barrier protecting the integrity of the cell membrane at low temperatures.

Other resistance mechanisms developed by the bacterium have also been observed in this study, and involve effects in nucleotide catabolism from nucleic acids and recycling pathways, being energetically less costly than their *de novo* synthesis, are probably favored in the bacterium in periods of stress. Also, there were negatively regulated at 20°C, the enzymes encoded by the operon involved in the *de novo* biosynthesis of pyrimidine nucleotides (a 2.2-, 1.8-, 1.6-, and 1.8 decrease, respectively, for the aspartate carbamoyltransferase, the dihydroorotase, the carbamoyl-phosphate synthase large chain, and the dihydroorotate dehydrogenase) as well those enzymes responsible for purine nucleotides biosynthesis (1.7-fold downregulation for both the glutamine amidotransferase and the adenylosuccinate lyase) (Supplementary Table 1). By contrast, the adenine phosphoribosyltransferase involved in the salvage pathways of these nitrogenous bases was 2.5-fold positively regulated (Supplementary Table 1).

Finally, the increase in the machinery involved in protein translation represents one of the most upregulated proteins (14%) at 20°C (Figure 3), correlating with a need for newly synthesized proteins (membrane transporters, cell wall components), which are the critical biological process for cell viability.

### 3.5. Oxidative stress-related protein

Oxygen is considered one of the critical factors affecting the survival of anaerobic aerotolerant LAB. It is known that under cold stress conditions, oxygen solubility increases, generating an increase in reactive oxygen species (ROS), leading to oxidative stress (Tribelli and Lopez, 2018). When accumulated, ROS can lead to damage in proteins, DNA, and lipids and thus trigger the expression of specific proteins to protect cell machinery. In this context, a range of antioxidant enzymes was detected as overexpressed at 20°C (Supplementary Table 1), such as NADH oxidase (5.0-fold upregulation), glutathione peroxidase (3.4-fold upregulation), and a thiol peroxidase (1.7-fold upregulation). In addition, the glutathione reductase and other thiol peroxidase were only detected at 20°C (Supplementary Table 2), indicating that *Lc. lactis* AV1n is undergoing real oxidative stress at low temperatures. NADH oxidase is an O_2_-consuming enzyme, generally involved in the aerobic metabolism of microaerophilic bacteria, responsible for the rapid removal of O_2_, and plays a crucial role in maintaining the intracellular redox balance (Feng and Wang, 2020). Interestingly, another NADH oxidase that is involved in the electron transfer chain was only detected at 37°C, indicating that oxidative stress also takes place at this temperature.

In addition, the glutathione redox cycle allows the protection of the cells against oxidative stress as previously described for *Lactobacillus fermentum* ME-3 (Kullisaar et al., 2010). In this cycle, glutathione is oxidized by glutathione peroxidase to a disulfide, which can be reduced back to glutathione by glutathione reductase (Feng and Wang, 2020). In this context, *Lc. lactis* AV1n overproduces the two enzymes involved in the glutathione redox cycle at 20°C, a result supporting that the bacterium is subjected to oxidative stress at this temperature.

Moreover, the upregulation of the 3 DBRH at 20°C can be assigned to the presence of oxidative stress in addition to the cold response (Salze et al., 2020).

Furthermore, at 20°C, a 2.3-fold increase was observed in the demethylmenaquinone methyltransferase (Ac: A0A0Q0U6R9), an enzyme that catalyzes the final step of menaquinone (vitamin K_2_) biosynthesis. Also, at this low temperature, a 3.1-fold increase in cytochrome O ubiquinol oxidase (Ac: A0A1 × 0V2Y4) was observed, an enzyme involved in the synthesis of ubiquinone (coenzyme Q). Both vitamin K_2_ and coenzyme Q are part of the electron transport chain of aerobic respiratory metabolism, and although LAB has historically been classified as anaerobic, aerotolerant, and unable to obtain energy through respiration, a variety of them contain rudimentary electron transport chains that can be reconstituted by the addition of either heme alone or combined heme and menaquinone to the growth medium. Therefore, these activated electron transport chains at 20°C could lead to higher biomass production, provide a contribution to make the bacteria more resistant to oxidative and acidic stresses, and support prolonged survival at low temperatures through respiration (Brooijmans et al., 2009). It can, thus, be assumed that the upregulation of the synthesis of these molecules is clearly an additional defense against oxidative stress for *Lc. lactis* AV1n (Papadimitriou et al., 2016).

### 3.6. Behavior and survival of *Lc. lactis* AV1n[pRCR21] under cold shock

**FIGURE 9:**
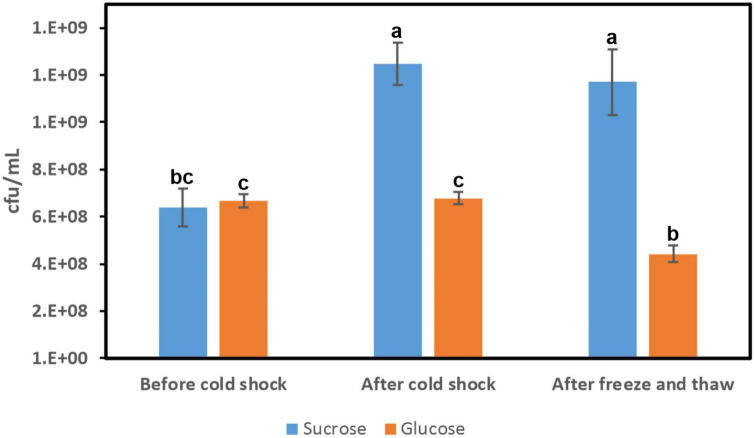
Influence of cold shock in *Lc. lactis* AV1n[pRCR21] cell viability. Cultures were grown in MRSS or MRSG at 37°C (before cold shock), after exposure at 10°C for 18 h (after cold shock), and during 3 days frozen period at −80°C, thawed (freeze and thaw). Cell survival was determined by plate counting at the states indicated. The means of three determinations and the standard deviations are indicated. Different superscript letters indicate that the levels differed significantly (*p* = 2e^–5^).

Previous studies have shown that, at extreme temperatures (e.g., −20°C), cell viability was significantly improved, when bacteria were previously cold shocked, allowing resistance mechanisms to be developed for cell adaptation to the new conditions (Kim and Dunn, 1997). Among these mechanisms, the results reported here indicated that the dextran produced by *Lc. lactis* could provide the bacterium protection of its cellular integrity against cold shock stress by constituting a physical barrier. To test this hypothesis, *Lc. lactis* AV1n[pRCR21] was subjected to a two-stage cold shock by: (1) an abrupt shift of exponentially growing cultures from 37°C to 10°C and incubation of the bacterium at this latter temperature for 18 h and (2) a subsequent transfer of the cultures at −80°C and their maintenance frozen during 3 days. These bacterial cold shocks were performed in the presence or absence of dextran production (bacterial cultures in MRSS or MRSG medium). Then, cell survival was assayed by plate counting prior to transfer of the cultures at 10°C, after the cold shock at 10°C, and after thawing the frozen cultures (Figure 9). Moreover, before and after the exposure to 10°C, the transcription of the *dsrLL* gene was estimated by measurement of the mCherry fluorescence emitted by the bacteria and expressed from the *mrfp* gene fussed to the P*_*dsrLL*_* promoter (Table 1). In addition, the levels of dextran produced during the 10°C cold shocks were determined.

**TABLE 1:** Analysis of fluorescence and growth of *Lc. lactis* AV1n[pRCR21] before and after 10°C cold shock.

Condition	Growth medium	Fluorescence (a.u.)	OD_600 nm_	^1^Ratio Fl/OD	^2^Ratio Fl/OD in MRSS Fl/OD in MRSG
After cold shock	MRSS	4.96 ± 0.20	8.35 ± 0.58	0.60 ± 0.03*^A^*	2.62*^a^*
Before cold shock	MRSS	3.43 ± 0.34	6.63 ± 0.25	0.54 ± 0.05*^A^*	2.73*^a^*
After cold shock	MRSG	1.61 ± 0.31	7.40 ± 0.75	0.22 ± 0.01*^B^*	
Before cold shock	MRSG	0.93 ± 0.60	4.43 ± 0.30	0.21 ± 0.14*^B^*	

The bacterium was grown at 37°C in either MRSS or MRSG and then subjected to 10°C cold shocks for 18 h. The means of three determinations and the standard deviations are indicated. ^1^The fluorescence (Fl) of the cultures was corrected taking in account the biomasses of the cultures estimated by the OD_600 nm_ (OD) and calculated as the ratio Fl/OD. Different superscript letters indicate that the levels differed significantly (*p* ≤ 2e^–3^). Differences between “before cold” in MRSS and MRSG (*p* = 2e^–3^) and between “after cold” in MRSS and MRSG (*p* = 8e^–6^) were significant. ^2^The Influence of growth medium in levels of fluorescence was estimated by calculating the ratio of Fl/OD.

Comparison of the mCherry fluorescence from cultures grown in MRSS and MRSG revealed that before and after the cold shock, the presence in the medium of sucrose, instead of glucose, resulted in a 2.7- and 2.6-fold increase in transcription from the P*_*dsrLL*_* promoter, supporting previous results obtained when testing the influence of carbon source in cultures grown at 37°C or 20°C (Besrour-Aouam et al., 2019). In addition, a low and not significant (*p* = 0.09) increase in fluorescence was observed corrected for the biomass after the temperature downshifts in both growth media (in MRSS, 0.60 ± 0.03 after vs. 0.54 ± 0.05 before the cold shock, as well as in MRSG 0.22 ± 0.01 after vs. 0.21 ± 0.14 before cold shock), showing that the bacterium exposure to 10°C for 18 h did not result in high activation of transcription from P*_*dsrLL*_*. The interaction between “Before cold and After cold” in either MRSS or MRSG was not significant (*p* = 0.09). However, the production of dextran was detected (0.33 ± 0.06 g/L) during the 18 h incubation at 10°C in MRSS, showing that the DsrLL was, in fact, active in this condition since the dextran previously produced by the bacterium at 37°C was removed by sedimentation and resuspension of the cells in fresh MRSS medium prior to the cold shock treatment.

In general, upon temperature downshift, there is a transient arrest of bacterial cell growth for a few hours called the acclimation phase when the bacteria adapt to the new environment by producing cold-inducible proteins. After this phase, cells become adapted to low temperatures and resume growth but at a slower rate (Barria et al., 2013). Analysis of cell survival of *Lc. lactis* AV1n[pRCR21] revealed better adaptation of the bacterium to the cold shock in MRSS than in MRSG (Figure 9). In fact, in the presence of glucose cells, growth did not take place during the 18 h incubation period at 10°C since the number of viable cells did not increase (6.67 × 10^8^ ± 0.29 × 10^8^ cfu/ml before vs. 6.78 × 10^8^ ± 0.25 × 10^8^ cfu/ml after cold shock) (Figure 9). However, in the presence of sucrose, the bacterium grew during the 10°C cold shocks, and the number of viable cells increased 2-fold, with statistical significance (*p* = 2e^–5^), from 6.38 × 10^8^ ± 0.81 × 10^8^ to 1.25 × 10^9^ ± 0.90 × 10^8^ cfu/ml (Figure 9). According to the results presented here, it seems that the acclimation phase for *Lc. lactis* AV1n took place during the 18 h at 10°C in the presence of sucrose and not of glucose, and this difference could be partially attributed to the production of dextran by the strain during growth at 10°C in MRSS, which should support better adaptation to the cold temperature.

We also observed that cell viability after 3 days of freezing at −80°C was better for cold-pretreated cultures in MRSS (93.6% survival) than those in MRSG (65.2% survival) (Figure 9), supporting also the potential cryoprotective role of the dextran produced by the bacterium. In fact, this activity has already been demonstrated for HePS produced by other bacteria, e.g., the polymer synthesized by *Pseudomonas* sp. ID1, which was able to preserve bacterial cell structure and contribute to the maintenance of bacterial viability after exposure to freezing temperature (Carrión et al., 2015). Also, the HePS from *Colwellia psychrerythraea* 34H showed a cryoprotection effect by having a significant inhibitory effect on ice recrystallization (Casillo et al., 2017). However, it should be stated that dextran formation is a multifactorial extracellular process and the amount of dextran may also arise from other factors and not necessarily from higher enzyme concentration, e.g., slower extracellular acidification during growth at low temperature. Therefore, further studies are needed to clarify the cryoprotectant potential effect of the dextran.

## 4. Conclusion

From the proteomic analysis performed here, it can be inferred that the cold adaptation mechanism used by *Lc. lactis* AV1n is mainly based on an improvement of its protein synthesis capacity, an increase in dextran production, and peptidoglycan biosynthesis in order to protect the cells from external aggression. It also consists of an energy saving by the decrease in the growth rate mediated not only by the decrease in the carbohydrate metabolism and its orientation toward the production of storage molecules but also by the preferential use of molecule recycling pathways, rather than their *de novo* synthesis.

The adaptation of LAB to low temperatures contributes to the industrial performance of the strains. Thus, a better understanding of reactions at low temperatures can contribute to the optimization of fermentation processes at cold temperatures, refrigerated storage of fermented products, and conservation conditions of starter cultures, thus enhancing the biotechnological potential of strains for industrial applications. Therefore, the knowledge gained from this work allowed a better understanding of the role of DsrLL in the response of *Lc. lactis* to low temperature and it could contribute to a better future use of strains of the genus *Leuconostoc* as probiotics for the development of new functional foods. Moreover, this work by the *in vivo* and *in silico* analysis of DsrLL expression should provide a better understanding of the complex regulation of gene expression at low temperatures in *Lc. lactis.*

## Data availability statement

The datasets presented in this study are available at the following link: https://www.ebi.ac.uk/pride/archive/projects/PXD037250.

## Author contributions

NB-A contributed to all parts of the experimental work and wrote a draft of the manuscript. VL performed the proteomic analysis. MM designed the experiments and interpreted the results. AH-A and AN contributed to the study design. PL participated in the study conception, data interpretation, and generated the final version of the manuscript. H-IO participated in study conception, data interpretation, and manuscript revision. All authors have read and approved the final manuscript.

## Footnotes

1
http://www.uniprot.org/

2
https://www.genome.jp/kegg/pathway.html
