# Supplementary material for: Proteomic and in silico analyses of dextran synthesis influence on Leuconostoc lactis AV1n adaptation to temperature change
Source: Front Microbiol. 2023 Jan 11;13:1077375. doi: 10.3389/fmicb.2022.1077375 (PMC9875047; doi:10.3389/fmicb.2022.1077375)
Supplement: Supplementary file 1 [file Table_1.docx]

Supplementary Material

**Table S1. *Lc*. *lactis* AV1 [pRCR21] proteins with different levels when grown in MRSS at 20 °C or 37 °C.**

| **Name** | **Accession number** | **^1^Fold change**  **20 ºC vs 37 ºC** | **Up- or**  **Downregulated** |
| --- | --- | --- | --- |
| Phosphonate ABC transporter substrate-binding protein | A0A1B2A136 | 52.93662 | Up |
| Cold-shock protein CspA | A0A0N8VZ44 | 39.91730 | Up |
| Uncharacterized protein | A0A1B2A101 | 25.87027 | Up |
| Acetyltransferase | A0A1B2A1R7 | 19.51324 | Up |
| Uncharacterized protein | A0A1B2A100 | 10.16175 | Up |
| Phosphonate ABC transporter substrate-binding protein | A0A0Q0Z1H6 | 9.92723 | Up |
| Flavodoxin | A0A1X0V3K5 | 8.46103 | Up |
| Glycosyl transferase | A0A1B2A353 | 8.35599 | Up |
| [Ribosomal protein S18]-alanine N-acetyltransferase | A0A1B2A1E8 | 7.97729 | Up |
| ABC transporter permease | A0A0N8VYI6 | 7.19130 | Up |
| Nucleoside diphosphate kinase | A0A1X0V0L1 | 6.72319 | Up |
| Peptide ABC transporter substrate-binding protein | A0A1B2A105 | 6.51885 | Up |
| Fructokinase | A0A1B2A288 | 6.20503 | Up |
| Protein mCherry | A0A0Q00AJ0 | 6.17528 | Up |
| ABC transporter ATP-binding protein | A0A1B2A0V9 | 5.93334 | Up |
| PTS sucrose transporter subunit EIIBCA | A0A1X0V2U8 | 5.90041 | Up |
| Energy-coupling factor ABC transporter ATP-binding protein | A0A1B2A1K6 | 5.63352 | Up |
| Adenine DNA glycosylase | A0A1B2A0F | 5.24170 | Up |
| NADH oxidase | A0A1B2A2Q7 | 5.05173 | Up |
| Pyridoxal 5-phosphate synthase subunit PdxS | A0A0Q1A2A1 | 4.78273 | Up |
| Transcriptional regulator | A0A1B1ZZU1 | 4.56147 | Up |
| RNA-binding transcriptional accessory protein | A0A1B2A2R9 | 4.38792 | Up |
| Glutathione peroxidase | A0A0Q0U5X4 | 4.35450 | Up |
| NADPH:quinone reductase | A0A1X0V325 | 4.25769 | Up |
| Glyoxalase | A0A1B1ZZY8 | 4.16796 | Up |
| DUF805 domain-containing protein | A0A1B2A2E5 | 4.11686 | Up |
| 2-amino-4-hydroxy-6-hydroxymethyldihydropteridine diphosphokinase | A0A1X0UZV3 | 4.05878 | Up |
| Pyridoxal 5-phosphate synthase subunit PdxT | A0A1X0V2M0 | 3.71561 | Up |
| ATP-dependent RNA helicase CshA | A0A1B1ZZX1 | 3.70038 | Up |
| Uncharacterized protein | A0A1X0V1J7 | 3.67381 | Up |
| Diguanylate cyclase | A0A1X0V3V0 | 3.66344 | Up |
| Primosomal protein N | A0A1X0V3R6 | 3.64411 | Up |
| CCA-adding enzyme | A0A0Q0TS06 | 3.61626 | Up |
| Pseudouridine synthase | A0A0Q0Y9W8 | 3.60475 | Up |
| Sortase | A0A1X0V1E5 | 3.53877 | Up |
| Serine hydrolase | A0A1X0V223 | 3.47693 | Up |
| Membrane protein insertase YidC | A0A0Q0YF52 | 3.45337 | Up |
| Glutathione reductase | A0A1X0V483 | 3.42197 | Up |
| Amino acid permease | A0A0Q1DSB1 | 3.36240 | Up |
| Carboxylesterase | A0A1B2A020 | 3.20487 | Up |
| Uncharacterized protein | A0A1X0V0S3 | 3.16399 | Up |
| Crp/Fnr family transcriptional regulator | A0A1B2A0S7 | 3.14632 | Up |
| Cytochrome O ubiquinol oxidase | A0A1X0V2Y4 | 3.12990 | Up |
| Aspartate ammonia-lyase | A0A1X0V348 | 3.12470 | Up |
| tRNA dimethylallyltransferase | A0A1X0V3T9 | 3.07506 | Up |
| 5-methylthioadenosine/S-adenosylhomocysteine nucleosidase | A0A0Q0U5Q2 | 3.05288 | Up |
| ADP-ribose pyrophosphatase | A0A1B2A2Y5 | 2.96086 | Up |
| Oxidoreductase | A0A1B2A2L5 | 2.94797 | Up |
| Uncharacterized protein | A0A1X0V1P2 | 2.92770 | Up |
| Acetylornithine transaminase | A0A1B2A0Y2 | 2.92693 | Up |
| ADP-ribose pyrophosphatase | A0A1B2A280 | 2.90220 | Up |
| Uncharacterized protein | A0A0Q0Y622 | 2.89273 | Up |
| Signal peptidase I | A0A1X0V2C5 | 2.86725 | Up |
| NADPH:quinone reductase | A0A1B2A0J1 | 2.85302 | Up |
| DNA-entry nuclease | A0A1B2A032 | 2.80653 | Up |
| Mini-ribonuclease HIII | A0A1X0V0Y9 | 2.78775 | Up |
| Zn-dependent peptidase | A0A0N8VZ10 | 2.75430 | Up |
| Zn-dependent peptidase | A0A1B2A206 | 2.69194 | Up |
| RNA-binding protein | A0A1B1ZZ31 | 2.69079 | Up |
| NAD(P)-dependent oxidoreductase | A0A1X0V111 | 2.61518 | Up |
| Nucleoside hydrolase | A0A1B1ZYZ3 | 2.56641 | Up |
| Hemolysin | A0A1B2A155 | 2.50640 | Up |
| Probable cell division protein WhiA | A0A0Q1DLR8 | 2.50131 | Up |
| 3-oxoacyl-ACP reductase | A0A1B1ZYY4 | 2.49856 | Up |
| RNA methyltransferase | A0A0Q0YER7 | 2.48730 | Up |
| Adenine phosphoribosyltransferase | A0A0Q0U697 | 2.47319 | Up |
| DNA polymerase I | A0A1B2A2A3 | 2.46976 | Up |
| 23S rRNA (Uracil-5-)-methyltransferase RumA | A0A1B2A1R1 | 2.43268 | Up |
| Cell division protein FtsW | A0A1X0V3U3 | 2.42844 | Up |
| Substrate-binding protein | A0A1B2A1N6 | 2.41511 | Up |
| N5-carboxyaminoimidazole ribonucleotide synthase | A0A1X0V3E0 | 2.38425 | Up |
| Acetolactate synthase | A0A1B2A1N1 | 2.38224 | Up |
| Alpha-acetolactate decarboxylase | A0A1X0V3U4 | 2.38140 | Up |
| Pyridine nucleotide-disulfide oxidoreductase | A0A1B2A0P2 | 2.37627 | Up |
| Butanol dehydrogenase | A0A1B2A2P7 | 2.37548 | Up |
| Ribonuclease M5 | A0A0Q0U6B5 | 2.31630 | Up |
| Queuine tRNA-ribosyltransferase | A0A0Q0TQJ4 | 2.30358 | Up |
| Demethylmenaquinone methyltransferase | A0A0Q0U6R9 | 2.29504 | Up |
| Glutamine ABC transporter substrate-binding protein | A0A1B2A017 | 2.29477 | Up |
| TetR family transcriptional regulator | A0A1B2A1B7 | 2.27381 | Up |
| RNA methyltransferase | A0A1B1ZZT6 | 2.25448 | Up |
| 50S ribosomal protein L11 | A0A0Q0Z133 | 2.25159 | Up |
| Undecaprenyl-diphosphatase | A0A0Q0U6T2 | 2.25131 | Up |
| GNAT family N-acetyltransferase | A0A1X0V3X0 | 2.24580 | Up |
| 50S ribosomal protein L14 | A0A0Q0U9V9 | 2.21838 | Up |
| Glutamine synthetase | A0A1X9VPH5 | 2.21155 | Up |
| Aminodeoxychorismate synthase, component I | A0A1X0V0Q0 I | 2.18072 | Up |
| Aspartate aminotransferase | A0A1B1ZYR6 | 2.17770 | Up |
| 50S ribosomal protein L17 | A0A0Q1A811 | 2.17690 | Up |
| 50S ribosomal protein L30 | A0A1X0V5B0 | 2.16862 | Up |
| Sugar transporter | A0A0Q0Y7P2 | 2.16272 | Up |
| Lipoprotein | A0A1B2A1N2 | 2.15764 | Up |
| Acetyltransferase | A0A1B2A0U8 | 2.14415 | Up |
| 50S ribosomal protein L4 | A0A1B2A111 | 2.09919 | Up |
| 2-succinyl-5-enolpyruvyl-6-hydroxy-3-cyclohexene-1-carboxylate synthase | A0A1X0V1Q5 | 2.09111 | Up |
| Gluconate kinase | A0A1B1ZZS2 | 2.06393 | Up |
| Cell division protein FtsK | A0A1B1ZZD1 | 2.05416 | Up |
| 50S ribosomal protein L23 | A0A0Q0Z0X5 | 2.03088 | Up |
| Cyclic-di-AMP phosphodiesterase | A0A1B2A0J8 | 2.01076 | Up |
| 30S ribosomal protein S9 | A0A0Q0U6H5 | 1.99392 | Up |
| Peptidoglycan hydrolase | A0A0Q0YGL5 | 1.99295 | Up |
| 3-oxoacyl-[acyl-carrier-protein] synthase 2 | A0A1B2A029 | 1.98473 | Up |
| Uncharacterized protein | A0A1X0V131 | 1.98188 | Up |
| UDP-N-acetylmuramoyl-tripeptide--D-alanyl-D-alanine ligase | A0A1B1ZZX0 | 1.97726 | Up |
| Small ribosomal subunit biogenesis GTPase RsgA | A0A1B1ZZK8 | 1.97542 | Up |
| MarR family transcriptional regulator | A0A0Q0YFY4 | 1.96460 | Up |
| Acetoin reductase | A0A0Q0U1J2 | 1.96038 | Up |
| Single-stranded-DNA-specific exonuclease RecJ | A0A1X0V2W2 | 1.95694 | Up |
| DNA-binding response regulator | A0A1B2A1V6 | 1.94306 | Up |
| Gluconate permease | A0A1B2A0V6 | 1.93990 | Up |
| tRNA-dihydrouridine synthase | A0A1B2A2I1 | 1.93245 | Up |
| Transcriptional regulator | A0A1X0V138 | 1.92843 | Up |
| Uncharacterized protein | A0A1B2A2S1 | 1.92667 | Up |
| Cell division protein | A0A1B1ZZG7 | 1.91721 | Up |
| 50S ribosomal protein L2 | A0A1B2A138 | 1.90606 | Up |
| Glycosyl transferase | A0A1X0V2Q6 | 1.90505 | Up |
| 2-succinylbenzoate--CoA ligase | A0A1X0V2B7 | 1.90119 | Up |
| ABC transporter ATP-binding protein | A0A1X0V2H5 | 1.90007 | Up |
| 50S ribosomal protein L18 | A0A0N8VZ40 | 1.89416 | Up |
| Holliday junction ATP-dependent DNA helicase RuvA | A0A1X0V2X3 | 1.89031 | Up |
| 50S ribosomal protein L13 | A0A0Q0YK79 | 1.88975 | Up |
| Pseudouridine synthase | A0A1B2A338 | 1.88544 | Up |
| Ribonuclease J1 | A0A1B2A1N5 | 1.88049 | Up |
| 30S ribosomal protein S16 | A0A1B2A214 | 1.88030 | Up |
| Mini-ribonuclease 3 | A0A0Q0YK89 | 1.87422 | Up |
| dITP/XTP pyrophosphatase | A0A0Q0Z0F6 | 1.86746 | Up |
| Alkaline phosphatase | A0A1X0V0Z4 | 1.83468 | Up |
| Type I restriction endonuclease subunit R | A0A1B1ZZE9 | 1.82333 | Up |
| Probable GTP-binding protein EngB | A0A0N8VYG5 | 1.80336 | Up |
| Phosphoesterase | A0A0Q0U9G9 | 1.79393 | Up |
| 50S ribosomal protein L21 | A0A0N8VYR4 | 1.79251 | Up |
| GNAT family N-acetyltransferase | A0A1X0V4J7 | 1.78381 | Up |
| ABC transporter ATP-binding protein | A0A0Q0YEX1 | 1.77508 | Up |
| DEAD-box ATP-dependent RNA helicase CshB | A0A1X0V2P8 | 1.77401 | Up |
| Phosphoglucosamine mutase | A0A1B2A1W3 | 1.77365 | Up |
| 1,4-dihydroxy-2-naphthoyl-CoA synthase | A0A1B2A0V0 | 1.77266 | Up |
| Thymidine kinase | A0A0Q0U6Q8 | 1.76719 | Up |
| Histidine kinase | A0A0Q0TQU0 | 1.76663 | Up |
| ACP phosphodiesterase | A0A0Q1DN29 | 1.75554 | Up |
| Peptidyl-prolyl cis-trans isomerase | A0A1X0V3L1 | 1.74785 | Up |
| Phosphate acyltransferase | A0A1B1ZZS1 | 1.74359 | Up |
| 50S ribosomal protein L5 | A0A0Q1DS25 | 1.73661 | Up |
| Phosphate import ATP-binding protein PstB | A0A1B2A216 | 1.73556 | Up |
| 50S ribosomal protein L15 | A0A0Q0TVM2 | 1.72021 | Up |
| Multidrug ABC transporter ATP-binding protein | A0A1B2A0P3 | 1.71674 | Up |
| Protein translocase subunit SecA | A0A1X9VQY5 | 1.70687 | Up |
| Thiol peroxidase | A0A0Q0TPS5 | 1.70315 | Up |
| Protein translocase subunit SecY | A0A1X0V189 | 1.69720 | Up |
| 30S ribosomal protein S20 | A0A1X0UWD3 | 1.69595 | Up |
| 30S ribosomal protein S8 | A0A0Q0YAQ2 | 1.68532 | Up |
| 30S ribosomal protein S10 | A0A1X0V560 | 1.68166 | Up |
| Phosphomethylpyrimidine kinase | A0A0Q1A5X3 | 1.67968 | Up |
| Single-stranded DNA-binding protein | A0A1B2A0H8 | 1.66771 | Up |
| ABC transporter permease | A0A1B1ZZA2 | 1.65148 | Up |
| Saccharopine dehydrogenase | A0A1B2A040 | 1.65039 | Up |
| EDD domain protein | A0A1B2A0Z6 | 1.64937 | Up |
| Thymidylate kinase | A0A1X0V0F9 | 1.64878 | Up |
| UDP-N-acetylenolpyruvoylglucosamine reductase | A0A1B2A1S7 | 1.64572 | Up |
| 50S ribosomal protein L3 | A0A0Q0TVK9 | 1.64317 | Up |
| 16S rRNA (Cytosine(967)-C(5))-methyltransferase | A0A1B1ZZN2 | 1.64315 | Up |
| Ribosomal silencing factor RsfS | A0A0Q0YV21 | 1.63245 | Up |
| 30S ribosomal protein S5 | A0A0Q0U6G2 | 1.62073 | Up |
| 1,4-dihydroxy-2-naphthoate prenyltransferase | A0A1X0V443 | 1.61677 | Up |
| Glutamyl aminopeptidase | A0A1B1ZZK7 | 1.60946 | Up |
| Uncharacterized protein | A0A1B2A064 | 1.59562 | Up |
| Type I pantothenate kinase | A0A1B2A0Z0 | 1.59153 | Up |
| Ribosomal RNA small subunit methyltransferase A | A0A1B2A1N4 | 1.59120 | Up |
| Exopolysaccharide biosynthesis protein | A0A1B2A2B8 | 1.58796 | Up |
| Uncharacterized protein | A0A1B2A1V0 | 1.58708 | Up |
| 50S ribosomal protein L16 | A0A0N8VZ39 | 1.55594 | Up |
| DNDP-4-keto-6-deoxy-glucose-2,3-dehydratase | A0A1B2A222 | 1.55571 | Up |
| Isochorismate synthase | A0A1B2A396 | 1.54617 | Up |
| Pyrroline-5-carboxylate reductase | A0A1B2A1D6 | 1.54573 | Up |
| Chemotaxis protein | A0A1B2A1J7 | 1.54272 | Up |
| HAD family hydrolase | A0A1B1ZZT4 | 1.53532 | Up |
| NADH dehydrogenase | A0A1B2A161 | 1.53357 | Up |
| ATP-dependent RNA helicase YfmL | A0A1X0V093 | 1.53001 | Up |
| Histidinol-phosphate aminotransferase | A0A1B2A1Y5 | 1.52794 | Up |
| Hydroxymethylglutaryl-CoA reductase | A0A1B2A088 | 1.51769 | Up |
| Acetyl-coenzyme A carboxylase carboxyl transferase subunit beta | A0A0Q0YFX8 | 1.51597 | Up |
| 30S ribosomal protein S7 | A0A0Q0Z0W8 | 1.51552 | Up |
| Uncharacterized protein | A0A1B2A0E9 | 1.50590 | Up |
| Redox-sensing transcriptional repressor Rex | A0A0Q0U6N1 | 1.50417 | Up |
| Aminotransferase | A0A1B1ZZB3 | 1.50034 | Up |
| UDP-galactopyranose mutase | A0A0N8VYY1 | 1.50019 | Up |
| Type I restriction enzyme R Protein | A0A1B2A2W8 | 0.66578 | Down |
| BMP family ABC transporter substrate-binding protein | A0A0Q1DL57 | 0.66507 | Down |
| DNA methyltransferase | A0A0Q0U4U1 | 0.66434 | Down |
| ATP synthase gamma chain | A0A1B2A0Y8 | 0.65937 | Down |
| Nicotinate phosphoribosyltransferase | A0A0Q0U5F1 | 0.65934 | Down |
| Nicotinate phosphoribosyltransferase | A0A1X0V320 | 0.65913 | Down |
| 2,3-bisphosphoglycerate-dependent phosphoglycerate mutase | A0A0Q0U9Y5 | 0.65316 | Down |
| DNA-binding response regulator | A0A0Q1A1Y7 | 0.65025 | Down |
| Lactate dehydrogenase | A0A1B2A0C8 | 0.64883 | Down |
| DNA repair protein RecN | A0A1B1ZZL1 | 0.64797 | Down |
| Uncharacterized protein | A0A1B1ZZ20 | 0.64723 | Down |
| UTP--glucose-1-phosphate uridylyltransferase | A0A1B2A201 | 0.64433 | Down |
| Shikimate dehydrogenase (NADP(+)) | A0A0N8VYJ4 | 0.63359 | Down |
| Amino acid ABC transporter ATP-binding protein | A0A1B2A0G7 | 0.62287 | Down |
| Protein GrpE | A0A1B2A3D8 | 0.61662 | Down |
| tr Carbamoyl-phosphate synthase large chain | A0A1X0V1Y4 | 0.61334 | Down |
| Glutamine ABC transporter ATP-binding protein | A0A0N8VYK9 | 0.61077 | Down |
| ADP-ribose pyrophosphatase | A0A1B2A0D5 | 0.61073 | Down |
| Serine/threonine protein phosphatase | A0A0Q1DMH1 | 0.60742 | Down |
| Peptidase T | A0A1B2A2Q0 | 0.60664 | Down |
| Dihydroorotate dehydrogenase | A0A1B2A2K3 | 0.60626 | Down |
| UPF0210 protein BCR17_07580 | A0A1B2A2L9 | 0.60072 | Down |
| Adenylyltransferase | A0A0Q0YVW4 | 0.59969 | Down |
| PTS mannose family transporter subunit IID | A0A1B2A3C0 | 0.59959 | Down |
| Uncharacterized protein | A0A1X0V047 | 0.59789 | Down |
| Glutamine amidotransferase | A0A1B2A1D1 | 0.59165 | Down |
| (P)ppGpp synthetase | A0A1B2A233 | 0.59062 | Down |
| Ribosome-recycling factor | A0A0Q0TTH4 | 0.58355 | Down |
| Adenylosuccinate lyase | A0A1B1ZZB2 | 0.58223 | Down |
| UDP-glucose 6-dehydrogenase | A0A1X9VSJ0 | 0.58167 | Down |
| Uncharacterized protein | A0A1B2A293 | 0.58079 | Down |
| Peptidoglycan-binding protein LysM | A0A1B2A076 | 0.57889 | Down |
| Diadenosine tetraphosphate hydrolase | A0A0Q0TRZ5 | 0.57608 | Down |
| Probable transcriptional regulatory protein AN225_08265 | A0A0Q0YUM1 | 0.57582 | Down |
| BMP family ABC transporter substrate-binding protein | A0A1X0V3C7 | 0.56683 | Down |
| PTS mannose transporter subunit EIIAB | A0A1B2A1F3 | 0.56668 | Down |
| Orotidine 5-phosphate decarboxylase | A0A0N8VYR8 | 0.56340 | Down |
| HD domain-containing protein | A0A1X0V1S0 | 0.56322 | Down |
| Pyridine nucleotide-disulfide oxidoreductase | A0A1B1ZYV7 | 0.56258 | Down |
| Two-component sensor histidine kinase | A0A1B2A1A4 | 0.56024 | Down |
| Endopeptidase | A0A1B2A0I4 | 0.55882 | Down |
| Phosphoglycerate kinase | A0A1B2A171 | 0.55828 | Down |
| Adenylate kinase | A0A0Q0Z0Z3 | 0.55624 | Down |
| Dihydroorotase | A0A1B2A2G4 | 0.55462 | Down |
| Gamma-glutamyl phosphate reductase | A0A1B2A046 | 0.55185 | Down |
| Cysteine--tRNA ligase | A0A1B2A151 | 0.54625 | Down |
| Uncharacterized protein | A0A1B2A2B2 | 0.54529 | Down |
| Glucose-6-phosphate isomerase | A0A0Q0U508 | 0.54376 | Down |
| Uncharacterized protein | A0A1X0V2L3 | 0.53830 | Down |
| 33 kDa chaperonin | A0A1B2A1J5 | 0.53498 | Down |
| NADH-dependent flavin oxidoreductase | A0A1B2A103 | 0.52937 | Down |
| Phosphoserine aminotransferase | A0A1B1ZZJ5 | 0.51636 | Down |
| Segregation and condensation protein B | A0A1X0V3K9 | 0.51327 | Down |
| ADP-dependent (S)-NAD(P)H-hydrate dehydratase | A0A0Q0YGB8 | 0.51260 | Down |
| Aminotransferase | A0A0Q0TPN7 | 0.50830 | Down |
| Uncharacterized protein | A0A1X0V471 | 0.50732 | Down |
| Uncharacterized protein | A0A1X0V0V7 | 0.49061 | Down |
| Heat-inducible transcription repressor HrcA | A0A1B2A2F1 | 0.48130 | Down |
| Thymidine kinase | A0A1B2A0C3 | 0.48063 | Down |
| Oxidoreductase | A0A0N8VY18 | 0.47725 | Down |
| Phosphate starvation-inducible protein PhoH | A0A1B1ZYZ6 | 0.47497 | Down |
| 3-phosphoglycerate dehydrogenase | A0A0Q0TSD3 | 0.47357 | Down |
| Peptidoglycan-binding protein LysM | A0A1X0V1C4 | 0.46782 | Down |
| Maltose phosphorylase | A0A1B1ZZ46 | 0.46442 | Down |
| Branched-chain-amino-acid aminotransferase | A0A1B2A2G1 | 0.46356 | Down |
| β-phosphoglucomutase | A0A1B1ZZA5 | 0.45652 | Down |
| Uncharacterized protein | A0A0Q0U2Z4 | 0.45137 | Down |
| Carbonic anhydrase | A0A1B2A0X2 | 0.44656 | Down |
| Uncharacterized protein | A0A1X9VP87 | 0.44182 | Down |
| Uncharacterized protein | A0A1B2A3E3 | 0.44071 | Down |
| 3-5 exonuclease YhaM | A0A1X0V3G4 | 0.44031 | Down |
| Serine protease | A0A1B2A0D6 | 0.43928 | Down |
| Ribose-5-phosphate isomerase A | A0A0Q0U1G4 | 0.43628 | Down |
| Aldo/keto reductase | A0A1X0V3K0 | 0.42982 | Down |
| Uncharacterized protein | A0A1B1ZZ98 | 0.42546 | Down |
| Sucrose operon repressor ScrR | A0A1B1ZZZ0 | 0.42047 | Down |
| Hypoxanthine phosphoribosyltransferase | A0A0Q0YWT3 | 0.41976 | Down |
| Uracil-DNA glycosylase | A0A1B1ZYU3 | 0.41515 | Down |
| Uncharacterized protein | A0A1B1ZZU0 | 0.41084 | Down |
| Aspartate carbamoyltransferase | A0A1B2A2I5 | 0.41009 | Down |
| Phosphoenolpyruvate carboxylase | A0A1B2A1M4 | 0.39325 | Down |
| Peptide ABC transporter ATP-binding protein | A0A1B2A317 | 0.38830 | Down |
| Glycerol transporter | A0A1X0V3R1 | 0.38452 | Down |
| X-Pro aminopeptidase | A0A1B2A2F2 | 0.35361 | Down |
| Copper oxidase | A0A1X0V032 | 0.35105 | Down |
| Aspartate-semialdehyde dehydrogenase | A0A1B2A0J6 | 0.34768 | Down |
| GntR family transcriptional regulator | A0A1B2A260 | 0.34382 | Down |
| Homoserine kinase | A0A0Q0TRW7 | 0.33606 | Down |
| ATP-dependent Clp protease proteolytic subunit | A0A0Q0YV31 | 0.33037 | Down |
| Potassium transporter Trk | A0A1B2A0U7 | 0.32919 | Down |
| DNA-binding response regulator | A0A1B2A1C1 | 0.32683 | Down |
| Alkaline phosphatase | A0A1B2A0S2 | 0.32603 | Down |
| Glucosamine-6-phosphate deaminase | A0A0Q1A354 | 0.31876 | Down |
| Probable transcriptional regulatory protein AN225_05420 | A0A0Q0U3G9 | 0.30058 | Down |
| Uncharacterized protein | A0A1B1ZZV5 | 0.30027 | Down |
| Phosphoglycerate dehydrogenase | A0A1B2A2G0 | 0.29791 | Down |
| Chaperone protein DnaK | A0A0Q0YZW2 | 0.29384 | Down |
| Universal stress protein UspA | A0A1B1ZZC6 | 0.26012 | Down |
| Aspartokinase | A0A1B1ZYX6 | 0.25308 | Down |
| Uncharacterized protein | A0A1B2A098 | 0.25034 | Down |
| Uncharacterized protein | A0A1B2A0P1 | 0.23916 | Down |
| Uncharacterized protein | A0A1B2A083 | 0.23551 | Down |
| N-acetylglucosamine-6-phosphate deacetylase | A0A1B2A398 | 0.23178 | Down |
| Homoserine dehydrogenase | A0A1B1ZYY7 | 0.22585 | Down |
| 5-formyltetrahydrofolate cyclo-ligase | A0A1B1ZZ01 | 0.22311 | Down |
| BMP family ABC transporter substrate-binding protein | A0A1B2A004 | 0.20130 | Down |
| Clp protease ClpE | A0A1B2A1U8 | 0.19818 | Down |
| LacI family transcriptional regulator | A0A1X0V3L0 | 0.19780 | Down |
| Cyclopropane-fatty-acyl-phospholipid synthase | A0A1B2A2S7 | 0.18988 | Down |
| Threonine synthase | A0A1B1ZYY5 | 0.18493 | Down |
| Universal stress protein UspA | A0A1B2A0D8 | 0.16317 | Down |
| Mannose-6-phosphate isomerase | A0A1X0V134 | 0.15799 | Down |
| 60 kDa chaperonin | A0A1B2A1I5 | 0.15626 | Down |
| Ribosome hibernation promoting factor | A0A1B2A1D0 | 0.15569 | Down |
| Peroxidase | A0A0Q0U9N6 | 0.15450 | Down |
| Uncharacterized protein | A0A1B2A1Q1 | 0.14890 | Down |
| Uncharacterized protein | A0A0Q0YZY0 | 0.14592 | Down |
| MFS transporter permease | A0A0Q0YA01 | 0.14119 | Down |
| ATP-dependent Clp protease ATP-binding subunit | A0A1B2A0H1 | 0.12864 | Down |
| 10 kDa chaperonin | A0A0Q0U6N9 | 0.12571 | Down |
| Transcriptional regulator | A0A1X0V340 | 0.10592 | Down |
| PTS mannose family transporter subunit IID | A0A1X0UUW6 | 0.08901 | Down |
| Uncharacterized protein | A0A1B2A310 | 0.08026 | Down |
| Uncharacterized protein | A0A1B1ZZ24 | 0.06544 | Down |

List of proteins, fold change values and accession numbers corresponding to the detected proteins are shown.

^1^Proteins are ordered taking into account the fold change, starting with the protein with the highest level at 20 ºC and finishing with the protein with the highest level at 37 ºC.

**Table S2. *Lc*. *lactis* AV1 [pRCR21] proteins only detected upon growth in MRSS at 20 ºC**

| **Name** | **Accession number** | **Category** |
| --- | --- | --- |
| Dextransucrase | A0A411G111 | EPS biosynthesis |
| Cold-shock protein CspA | A0A1B2A2G5 | Stress response |
| Glutathione reductase | A0A1B1ZYQ2 | Stress response |
| Thiol peroxidase | A0A1B2A0I8 | Stress response |
| IS30 family transposase | A0A1X9VTK5 | Processing of genetic information (DNA integration) |
| Phosphate ABC transporter substrate-binding protein | A0A1X0V102 | Membrane transport |
| AI-2E family transporter | A0A1X0V387 | Membrane transport |
| Amino acid ABC transporter substrate-binding protein | A0A1B2A1Z1 | Membrane transport |
| Phosphonates import ATP-binding protein PhnC | A0A1X0V2A7 | Membrane transport |
| Branched-chain amino acid transporter | A0A1X0V2C9 | Membrane transport |
| GntR family transcriptional regulator | A0A1B2A0P8 | Transcriptional regulator |
| LexA repressor | A0A1B2A2M1 | Transcriptional regulator |
| Hydrolase | A0A1X0V124 | General function prediction |
| Amidohydrolase | A0A1X9VP51 | General function prediction |
| Cys-tRNA(Pro)/Cys-tRNA(Cys) deacylase | A0A1B2A0E3 | Protein biosynthesis |
| Uncharacterized protein | A0A0Q1A3F8 | Uncharacterized |
| Uncharacterized protein | A0A1B1ZYX0 | Uncharacterized |
| Uncharacterized protein | A0A1B2A1P6 | Uncharacterized |
| Uncharacterized protein | A0A1X0V034 | Uncharacterized |

List of the detected proteins, Accession number and categories to which belong are shown.

**Table S3. *Lc*. *lactis* AV1 [pRCR21] proteins only detected upon growth in MRSS at 37 ºC**

| **Name** | **Accession number** | **Category** |
| --- | --- | --- |
| Clp protease ClpE | A0A1X9VPT9 | Stress response |
| DNA repair protein RecO | A0A1X0V1Q9 | Processing of genetic information |
| S-ribosylhomocysteine lyase | A0A0Q0TVT5 | Amino acid metabolism |
| 2,3-bisphosphoglycerate-dependent phosphoglycerate mutase | A0A1X0V3P9 | Carbohydrate metabolism |
| 6-pyruvoyl tetrahydropterin synthase | A0A1B2A1U9 | Cofactor metabolism |
| NADH oxidase | A0A1B2A1N3 | Electron transport chain |
| Lysophospholipase | A0A1X0V3B5 | Fatty acid metabolism |
| Uncharacterized protein | A0A1B2A0F6 | Uncharacterized |
| Uncharacterized protein | A0A0Q0U934 | Uncharacterized |

List of the detected proteins, Accession number and categories to which belong are shown.
